# Mechanochemical Synthesis of Fluorine-Containing Co-Doped Zeolitic Imidazolate Frameworks for Producing Electrocatalysts

**DOI:** 10.3389/fchem.2022.840758

**Published:** 2022-03-14

**Authors:** Max Rautenberg, Marius Gernhard, Jörg Radnik, Julia Witt, Christina Roth, Franziska Emmerling

**Affiliations:** ^1^ BAM Federal Institute of Materials Research and Testing, Berlin, Germany; ^2^ Department of Chemistry, Humboldt-Universität zu Berlin, Berlin, Germany; ^3^ Fakultät für Ingenieurwissenschaften, Lehrstuhl für Werkstoffverfahrenstechnik, Universität Bayreuth, Bayreuth, Germany

**Keywords:** MOF (Metal–Organic framework), mechanochemistry, XRD, electrocatalysis, mixed metal

## Abstract

Catalysts derived from pyrolysis of metal organic frameworks (MOFs) are promising candidates to replace expensive and scarce platinum-based electrocatalysts commonly used in polymer electrolyte membrane fuel cells. MOFs contain ordered connections between metal centers and organic ligands. They can be pyrolyzed into metal- and nitrogen-doped carbons, which show electrocatalytic activity toward the oxygen reduction reaction (ORR). Furthermore, metal-free heteroatom-doped carbons, such as N-F-Cs, are known for being active as well. Thus, a carbon material with Co-N-F doping could possibly be even more promising as ORR electrocatalyst. Herein, we report the mechanochemical synthesis of two polymorphs of a zeolitic imidazole framework, Co-doped zinc 2-trifluoromethyl-1H-imidazolate (Zn_0.9_Co_0.1_(CF_3_-Im)_2_). Time-resolved *in situ* X-ray diffraction studies of the mechanochemical formation revealed a direct conversion of starting materials to the products. Both polymorphs of Zn_0.9_Co_0.1_(CF_3_-Im)_2_ were pyrolyzed, yielding Co-N-F containing carbons, which are active toward electrochemical ORR.

## Introduction

Polymer membrane fuel cells (PMFCs) are of significant interest as a device for clean energy conversion. Their broad commercial application is currently limited by the inefficient oxygen reduction reaction (ORR). The slow kinetics of the ORR makes catalysis essential. State-of-the-art catalyst are currently based on platinum group metals (Gasteiger et al., 2005; Wu and Yang, 2013; Evers et al., 2019; Jiang et al., 2021; Zhang et al., 2021), but with their high price and scarcity, they limit a large-scale application. Therefore, low-cost and readily available alternatives are sought for. Reportedly, metal and nitrogen doped carbons (M-N-Cs) show high ORR activity, following the order M = Fe > Co > Mn > Cu >> Ni (Masa et al., 2014; Peng et al., 2014). The origin of the catalytic activity is speculated as M-N_4_ centers (M = Fe, Co) in a graphene matrix. However, Fe-based catalysts tend to produce Fe^2+/3+^ that can react with the ORR possible byproduct H_2_O_2_ generating hydroxyl and hydroperoxyl radical species. This mixture, known as Fenton’s reagent (Kang and Chang, 1997; Walling, 2002), can decompose organic matter, such as the proton conducting membrane of PMFCs. Alternatively, Co-based catalysts, showing similar performance as Fe-based catalysts without the risk of Fenton’s reaction, can be used.

**FIGURE 1:**
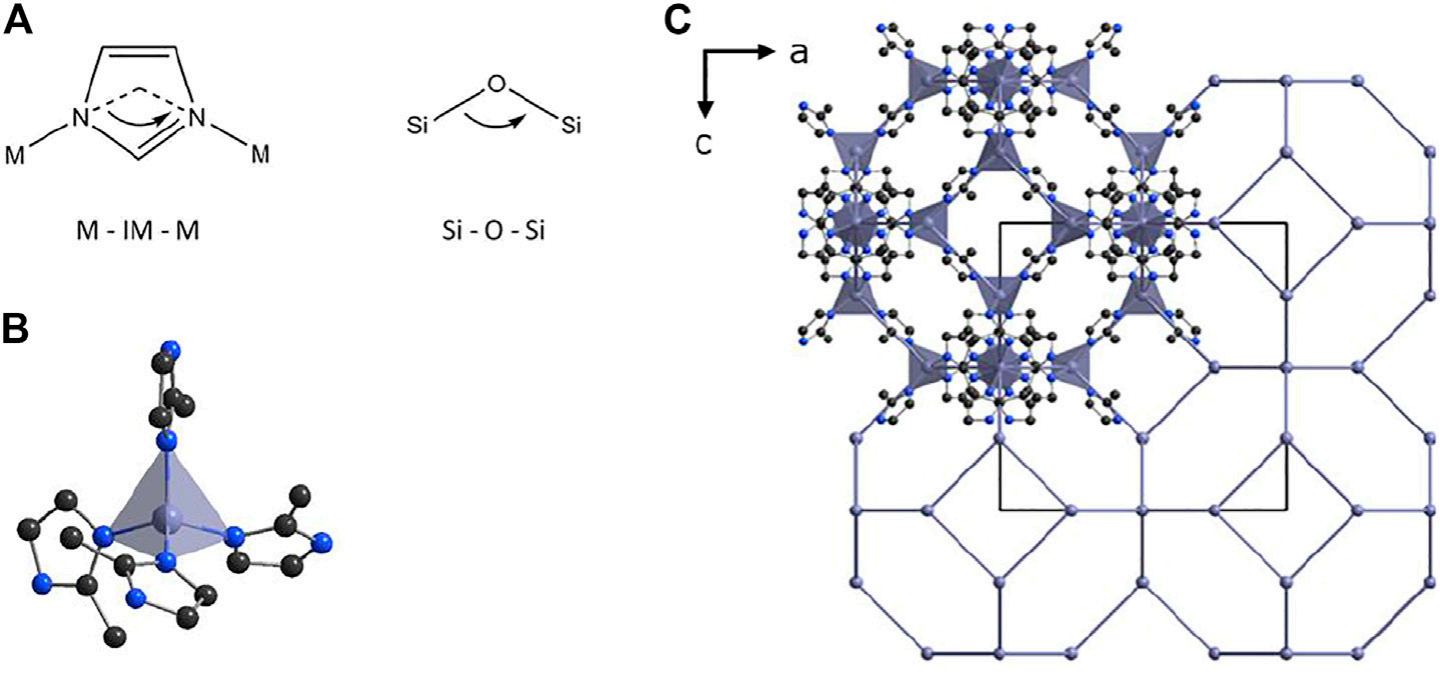
**(A)** The bond angle in zeolitic imidazolate frameworks (ZIFs) is similar to the Si-O-Si bond angle in zeolites. **(B)** In ZIF-8, each Zn^2+^ ion is tetrahedrally coordinated by four 2-methylimidazolate linkers. **(C)** View along the *b* axis of the crystal structure of ZIF-8. The top left unit is shown completely, the rest as a reduced net of Zn^2+^-ions, revealing the porous nature of the component.

Zeolitic imidazole frameworks (ZIFs) (Park et al., 2006), a class of metal organic frameworks (MOFs), consisting of metal nodes, linked by imidazole-based linkers can serve as templates, which can be converted in to M-N-C ORR catalysts by pyrolysis. ZIF materials are commonly used for gas storage (Eddaoudi et al., 2002) and separation (Keskin et al., 2010), oil spill cleaning (Mondal et al., 2017), catalysis (Farrusseng et al., 2009; Hu et al., 2020), sensing (Chapartegui-Arias et al., 2019; Zhang et al., 2020), and drug delivery (Hao et al., 2021; Ibrahim et al., 2017; Nirosha Yalamandala et al., 2021), as precursors for electrocatalysis (Li et al., 2016; Wang et al., 2014; Zhao et al., 2014) and as stimuli-responsive materials (Iacomi and Maurin, 2021). The crystal structures of these porous coordination polymers are based on the topology nets of zeolites. Furthermore, the metal-imidazolate–metal bond angles in ZIFs resemble the Si-O-Si bond angles in zeolites, as well as the tetrahedral coordination of metal centers by imidazolate ligands (Figures 1A,B). ZIFs have a broad variety of possible structures, depending on how the metal–imidazolate–tetrahedrons are interconnected (Schröder et al., 2013). In the well-studied compound ZIF-8, where zinc is tetrahedrally coordinated by 2-methylimidazolate linkers, the tetrahedral units form sodalite cages (SOD) (Figure 1C), resulting in a porous material (Park et al., 2006). Other ZIFs can form dense topologies, which resemble α-quartz (qtz).

Ma et al*.* showed that Co-based ZIFs can be easily pyrolyzed into Co-N-C catalysts for ORR by pyrolysis. (Ma et al., 2011). The resulting catalysts show high activity in alkaline media (Chao et al., 2015; Chen et al., 2015; Wang et al., 2016) but lower activity under acid conditions (Wang et al., 2014; You et al., 2015). Direct pyrolysis of cobalt 2-methylimidazolate (ZIF-67) leads to a porous carbon material with aggregated cobalt nanoparticles, resulting in a loss of active Co-N_4_ species and therefore ORR activity (Ma et al., 2011; Xia et al., 2014; Wang et al., 2016). Using zinc-based ZIFs with low amounts of cobalt results in a porous carbon material with uniformly distributed Co-N_4_ sites and no metallic cobalt particles, showing excellent ORR activity (Wang et al., 2016). Furthermore, doping of carbons with heteroatoms can yield ORR active materials. In case of N-, F-doping, a cooperative N and F polarization of carbon is speculated as the mechanism (Lv et al., 2017).

Different synthesis routes were established for ZIFs, including microwave-assisted (Bux et al., 2009), sonochemical (Son et al., 2008; Yang et al., 2012), electrochemical (Martinez Joaristi et al., 2012) and solvothermal methods (Palaniselvam et al., 2013), or crystal growth from solution (Venna et al., 2010). Most of these methods involve solvents such as N,N-dimethylformamide, N-N-diethylformamide, or methanol (Huang et al., 2006; Park et al., 2006; Cravillon et al., 2009; Kukkar et al., 2021). Mechanochemistry, as an alternative green and solvent-free method, has been established for several MOF synthesis including ZIF-8 (Pichon et al., 2006; Klimakow et al., 2010; Batzdorf et al., 2015; Chen et al., 2019; Szczęśniak et al., 2020).

Here, we present the mechanochemical synthesis of fluorine-substituted ZIF-8 frameworks including Co-doping. We figured a Co-doped Zn(CF_3_-Im)_2_ could be a promising precursor for a highly active ORR catalysts, combining positive effects of both CoN_4_-clusters and N-F-doping.

The samples were thoroughly characterized by X-ray diffraction (XRD), nitrogen sorption, transmission electron microscopy (TEM), and energy-dispersive X-ray spectroscopy (EDX). Furthermore, the formation process in the ball mill was followed by time-resolved *in situ* synchrotron XRD, gaining insights in the formation process of the compounds. The two polymorphs of Zn_0.9_Co_0.1_(CF_3_-Im)_2_ were pyrolyzed, and the carbonous materials’ chemical composition was analyzed by X-ray photoelectron spectroscopy (XPS). Furthermore, both pyrolyzed qtz- and SOD-Zn_0.9_Co_0.1_(CF_3_-Im)_2_ were shown to be active toward ORR.

## Experimental Section

### Materials

The following chemicals were used: zinc oxide (ZnO, ACS reagent; Acros Organics, USA), 2-methylimidazole C_4_H_6_N_2_ (≥98%; Sigma–Aldrich, Germany), cobalt (II) acetate tetrahydrate (Co(CH_3_COO)_2_ · 4 H_2_O; Baker analyzed, J. T. Baker, USA), zinc acetate (Zn(CH_3_COO)_2_ · 2 H_2_O; >98% ACS Reagent, Fluka; Honeywell International Inc.), basic zinc carbonate Zn_5_(CO_3_)_2_(OH)_6_ (>97%, Thermo Fisher Scientific, USA), 2-trifluoromethyl-1H-imidazole C_4_H_3_N_2_F_3_ (>95%; Fluorochem, United Kingdom), potassium hydroxide KOH (Sigma–Aldrich), perchloric acid HClO_4_ (Bernd Kraft, Germany) and isopropanol (Sigma–Aldrich). Nafion was purchased from Sigma–Aldrich. All chemicals were used without further purification.

### Synthesis of ZIF-8 and Zn_0.9_Co_0.1_(2Me-Im)_2_

Zinc oxide (0.337 mmol, 27.4 mg), zinc acetate dihydrate (10 mol% of total metal content, 0.037 mmol, 8.2 mg), 2-methylimidazole (0.748 mmol, 61.4 mg), and NH_4_NO_3_ (0.748 mmol, 3.0 mg) were placed into a custom-made milling jar (PMMA, 5 mL) (Lampronti et al., 2021). After adding one stainless-steel milling ball (7-mm diameter) and methanol (15 µL), the jar was closed and mounted into a vertical ball mill (Pulverisette 23; Fritsch GmbH, Idar-Oberstein, Germany). The mixture was ground for 15 min at a frequency of 50 Hz. The product was obtained as a white voluminous powder.

For Co-doping, the zinc acetate dihydrate was replaced by cobalt acetate tetrahydrate (10 mol% of total metal content, 0.037 mmol, 9.2 mg), which was added to the milling jar (PMMA, 5 mL), along with ZnO (0.333 mmol, 27.1 mg), 2-methylimidazole (0.740 mmol, 60.7 mg), NH_4_NO_3_ (0.037 mmol, 3.0 mg), methanol (15 µL), and a stainless-steel grinding ball (7-mm diameter). The mixture is ground for 15 min at a frequency of 50 Hz, and a purple voluminous powder is obtained.

### Synthesis of qtz-Zn(CF_3_-Im)_2_ and qtz-Zn_0.9_Co_0.1_(CF_3_-Im)_2_

In a typical experiment, hydrozincite (Zn_5_(CO_3_)_2_(OH)_6_, 0.052 mmol, 28.8 mg) and 2-trifluoromethyl-1H-imidazole (0.524 mmol, 71.3 mg) are weighed out and alongside a stainless-steel milling ball (7-mm diameter) are placed into a custom-made milling jar (PMMA, 5 mL). After adding methanol (15 µL), the jar was closed and mounted into a vertical ball mill (Pulverisette 23; Fritsch GmbH. The mixture was ground at a frequency of 50 Hz for 15 min. The product was obtained as a yellow–brown powder.

For Co-doping, the desired molar percentage of metal is replaced by cobalt acetate tetrahydrate. In a typical experiment with 10 mol% Co-doping, hydrozincite (Zn_5_(CO_3_)_2_(OH)_6_, 0.046 mmol, 25.0 mg), cobalt acetate tetrahydrate (10 mol% relative to total metal amount, 0.025 mmol, 6.2 mg), and 2-trifluoromethyl-1H-imidazole (0.505 mmol, 68.7 mg) are weighed out and placed into a custom-made milling jar (PMMA, 5 mL). After adding one stainless steel milling ball (7-mm diameter), the jar is closed and mounted into a (Pulverisette 23; Fritsch GmbH) vertical ball mill. The mixture was ground for 15 min at a frequency of 50 Hz. The product was obtained as a purple–brown powder.

### Synthesis of SOD-Zn(CF_3_-Im)_2_ and SOD-Zn_0.9_Co_0.1_(CF_3_-Im)_2_

To obtain SOD-Zn(CF_3_-Im)_2_ the reactant masses are kept constant (Zn_5_(CO_3_)_2_(OH)_6_: 0.052 mmol, 28.8 mg; 2-trifluoromethyl-1H-imidazole: 0.524 mmol, 71.3 mg), one stainless-steel milling ball (5-mm diameter) and DMF (20 µL) were used. The mixture was ground for 7 min at 50 Hz, and a damp brown powder was obtained. After completely drying the powder, it is washed three times with methanol (20 mL) and air dried.

Zn_0.9_Co_0.1_(CF_3_-Im)_2_ can be obtained when Zn_5_(CO_3_)_2_(OH)_6_ (0.046 mmol, 25.0 mg), cobalt acetate tetrahydrate (0.025 mmol, 6.3 mg), and 2-trifluoromethyl-1H-imidazol (0.505 mmol, 68.8 mg) are placed alongside a single grinding ball (5-mm diameter, stainless steel) into a custom-made milling jar (PMMA, 5 mL). The mixture is ground at 50 Hz for 8 min, yielding a damp purple–brown solid. After drying at air, the solid is finely ground in a mortar and washed with methanol (20 mL) three times and then dried at air.

### Preparation of Electrocatalysts

To prepare the electrocatalysts, the carbonous residue after carbonization was loaded on glassy carbon (GC) rotating disk electrode (RDE) according to the procedure described by Kocha et al. (2017). The method involved initial preparation of a stock solution with 10 mL isopropanol (Sigma–Aldrich), 0.2 mL of 5 wt% Nafion ionomer solution (Sigma–Aldrich) and 39.8 mL of deionized water (0.055 μS/cm, Evoqua, , United States). To prepare catalytic inks from the powder samples, 1.3 mg of the compound was mixed with 1 mL of the stock solution. The inks were homogenized for 45 min in an ultrasonic bath at 80 Hz. Afterward, the dispersion (10 µL) was deposited on a clean GC electrode and spun at 900 revolutions/min (rpm) until the liquid was evaporated.

Electrochemical characterizations of the heterogeneous catalyst powders were conducted using a three-electrode setup with a Gamry Reference 600 + potentiostat (Gamry Instruments, United States). Before each measurement, the electrolyte was degassed for 30 min with nitrogen and oxygen, respectively. All measurements were performed in 0.1 M KOH or in 0.1 M HClO_4_ by using a Pt counter electrode and an Ag/AgCl (3 M NaCl) reference electrode. Linear sweep voltammetry experiments were performed in a potential range of +1.1 V to −0.3 V in acidic media and +0.5 V to −0.8 V in alkaline media at a scan rate of 20 mV s^−1^, whereas the RDE was operated at rotation speeds of 600, 900, and 1,600 rpm. All potentials were reported with respect to the standard hydrogen electrode (SHE). The surface area of the GC electrode was 0.126 cm^2^. Prior to use, the GC electrode was polished with 0.3 and 0.05 mm alumina powder followed by sonicating and rinsing with deionized water after each polishing step for 5 min to remove the alumina and abraded particles.

### Powder XRD

Powder XRD data were collected using a Bruker D8 Advance diffractometer (Bruker AXS, Germany) in Bragg-Brentano-Geometry with a Lynxeye-detector using Cu-K_α_ radiation (*λ* = 1.542 Å) over a range of 2θ = 5°–60° with a step size of 0.02°. The time per step was 0.6 s. The finely ground dried sample was packed onto a standard PVC sample holder, which was mounted into the diffractometer.

### Synchrotron XRD

The *in situ* XRD experiments were performed at the μSpot beamline (BESSY II, Helmholtz Centre Berlin for Materials and Energy). The used beam diameter was 100 μm at a photon energy of 16.576 keV using a double crystal monochromator (Si 111). To minimize double reflections, the beam was positioned inside of the milling jar, by scanning the wall of the jar and then moving approximately 50 µm inside. The sample detector distance was 229.70 mm. Scattered intensities were collected with a two-dimensional X-ray detector (Eiger 9M, HPC 3,110 × 3,269 pixels, pixel size 75 × 75 µm) and a time-resolution of 30 s. The obtained scattering images were processed using an algorithm of the computer program DPDAK (Benecke et al., 2014). The resulting patterns (q/nm^−1^ vs. intensity/a.u.) were analyzed, processed, and plotted using Origin (Version 2020; OriginLabs Corporation, Northampton, MA, United States). For comparison, the theoretical XRD patterns of the starting materials and final products were retrieved from crystallographic databases ICSD or CCDC and simulated using Mercury (version 4.3.0, CCDC) (Macrae et al., 2020). All XRD plots are background corrected by a custom-made python script.

### Differential Scanning Calorimetry–TGA

Thermogravimetric analysis (TGA) and differential scanning calorimetry (DSC) was performed simultaneously on dry powders (∼10 mg) using a heat flux TGA-DSC 3+ (Mettler-Toledo). All measurements were carried out under a continuous nitrogen flow of 10 mL/min. As a reference, an empty α-Al_2_O_3_ corundum crucible was used. The samples were heated with a heating rate of 10 K/min from room temperature to 900°C and held for 1 h. Subsequently, the samples were allowed to cool down under continuous nitrogen gas flow.

### X-Ray Photoelectron Spectroscopy

All measurements were performed with an AXIS Ultra DLD photoelectron spectrometer manufactured by Kratos Analytical (Manchester, United Kingdom). XPS spectra were recorded using monochromatized aluminum Kα radiation for excitation, at a pressure of approximately 5 × 10^−9^ mbar. The electron emission angle was 0°, and the source-to-analyzer angle was 60°. The binding energy scale of the instrument was calibrated following a Kratos Analytical procedure, which uses ISO 15472 binding energy data. Spectra were taken by setting the instrument to the hybrid lens mode and the slot mode providing approximately a 300 × 700-μm^2^ analysis area. Furthermore, the charge neutralizer was used. Survey spectra were recorded with a step size of 1 eV and a pass energy of 80 eV; high-resolution spectra were recorded with a step size of 0.1 eV and a pass energy of 20 eV. Quantification was performed with Unifit 2021 using Scofield factor, the inelastic mean free pathway, and the transmission function for the normalization of the peak area. For peak fitting, a sum Gaussian–Lorentzian function was used. As background, a modified Tougaard background was used. Measurement uncertainties are ±0.2 eV with a confidence interval of 95% for binding energies at high-resolution spectra. Elemental quantification has a relative uncertainty of ±20% with a confidence interval of 95%.

### Transmission electron microscopy and Energy dispersive X-Ray Spectroscopy

TEM images were obtained in a Talos F200S Microscope (Thermo Fisher Scientific) by using a 200-kV microscopy technique in which a beam of electrons is transmitted through a specimen to form an image. The specimens were prepared by dropping sample solutions (1 mg/mL in water/solvent) onto a 3-mm copper grid (lacey, 400 mesh) and leaving them to air-dry at room temperature. To determine the elemental composition of the ZIF-8 and Zn_0.9_Co_0.1_ (2Me-Im)_2_ specimen, EDX with two silicon drift detectors (SDD) was used. Counting time for X-ray spectra was 60 s.

### Gas Sorption

Nitrogen gas sorption at 77 K was performed on an ASAP 2020 (Micrometrics) and was used to calculate the specific surface area from a multipoint adsorption isotherm with the BET (Brunauer–Emmit–Teller) calculation model (relative pressure range, 0.0012–0.0298) according to DIN ISO 9277:2014 (Brunauer et al., 1938).

## Results and Discussion

### Co-Doping of ZIF-8 by Acetate Ionic and Liquid-Assisted Grinding Route

**Scheme 1:**
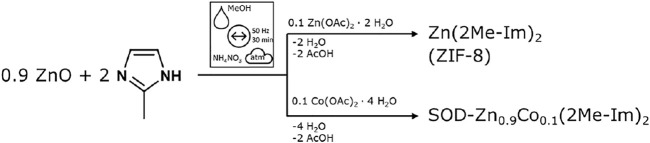
Scheme of the acetate ILAG route to obtain Zn (2Me-Im)_2_ (ZIF-8) and SOD-Zn_0.9_Co_0.1_ (2Me-Im)_2_, depending on the added metal acetate salt. The reaction conditions are summarized above the reaction arrow after ref. (Michalchuk et al., 2021).

**FIGURE 2:**
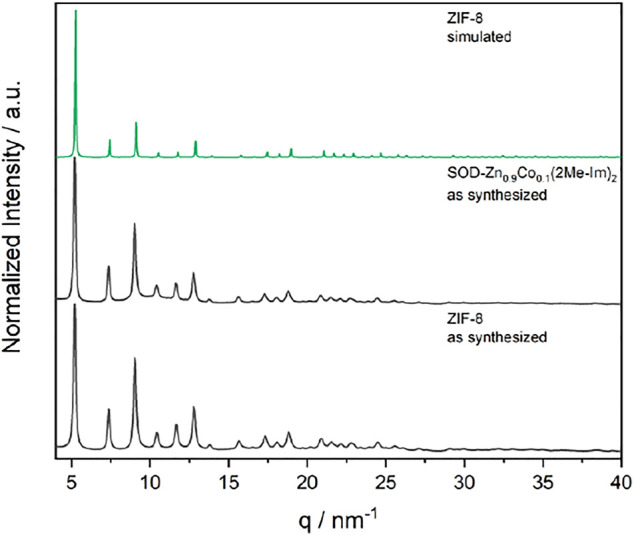
XRD patterns of as-synthesized ZIF-8 and SOD-Zn_0.9_Co_0.1_ (2Me-Im)_2_ by the acetate ILAG route (both black), both matching the simulated pattern of ZIF-8 (green).

Scheme 1 details the synthesis strategy to obtain ZIF-8 and SOD-Zn_0.9_Co_0.1_ (2Me-Im)_2_. We used a modified synthesis combining ionic and liquid-assisted grinding (ILAG) conditions (Friščić et al., 2010) and an acetate route described by Imawaka et al. (2019), Tanaka et al. (2017). Both ZIF-8 and SOD-Zn_0.9_Co_0.1_ (2Me-Im)_2_ were obtained phase pure and identified by XRD (Figure 2). All synthesis procedures were analyzed *via* time-resolved *in situ* XRD to analyze the reaction mechanism and potential phase transformations. These reactions were performed in a custom-built PMMA milling jar (Lampronti et al., 2021).

The SOD-Zn_0.9_Co_0.1_ (2Me-Im)_2_ powder was examined by TEM (Supplementary Figure S1) and EDX to assess its elemental composition. The Co content of 7.85% is close to the expected value of 10% of total metal content. Together with the XRD results, these data indicate the successful introduction of cobalt into the parental ZIF-8 structure (Supplementary Figure S2). Furthermore, the surface area of ZIF-8 and SOD-Zn_0.9_Co_0.1_ (2Me-Im)_2_ powders synthesized by the acetate ILAG route was studied after an activation protocol by nitrogen sorption at 77 K using the Brunauer–Emmett–Teller theory (Supplementary Figure S3). The samples exhibited type I isotherms with BET-surface areas of 1,695 m^2^/g (ZIF-8) and 1,554 m^2^/g (SOD-Zn_0.9_Co_0.1_ (2Me-Im)_2_), which are comparable to literature reports (Park et al., 2006; Kaur et al., 2016).

**FIGURE 3:**
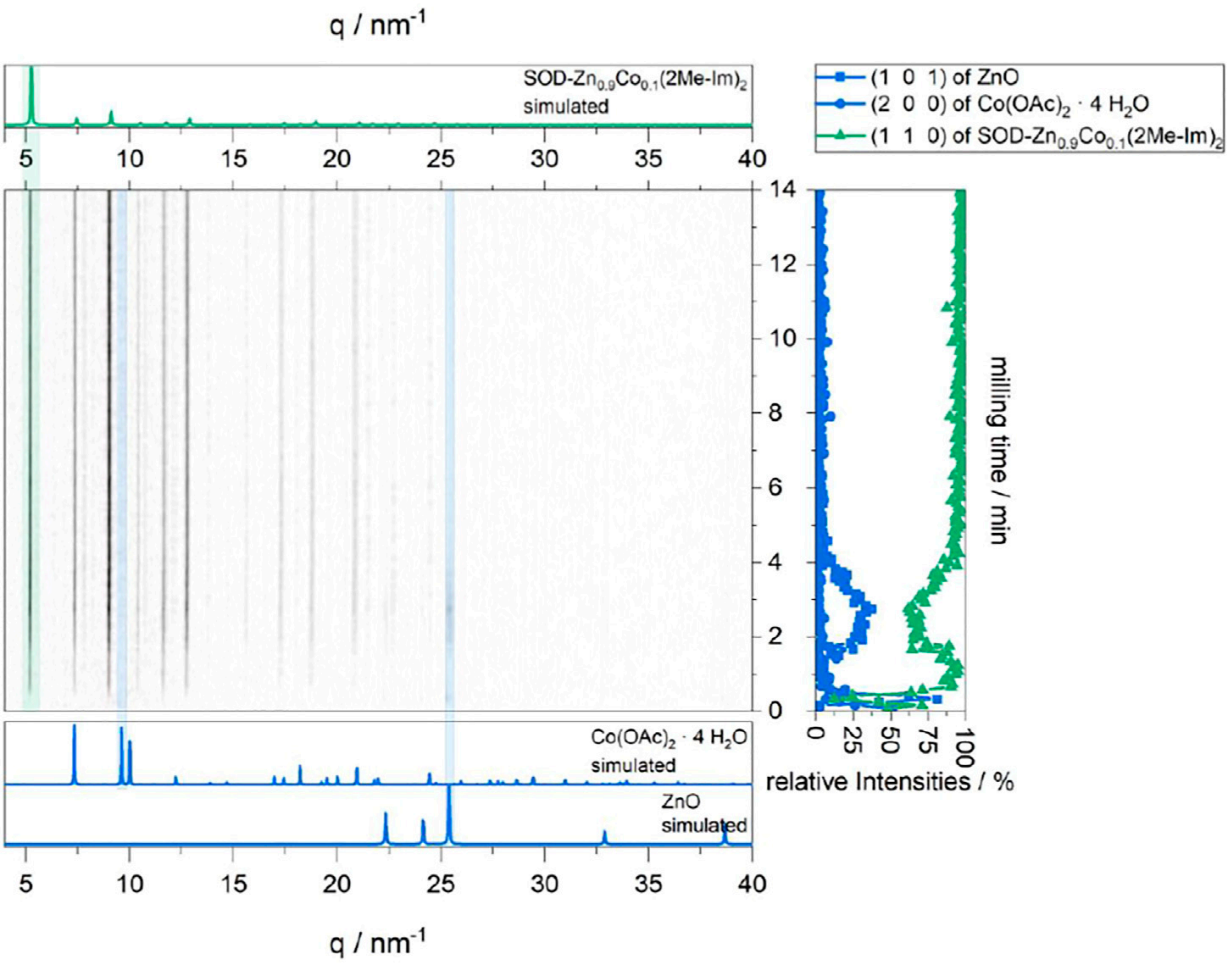
*In situ* XRD plot of the formation of SOD-Zn_0.9_Co_0.1_ (2Me-Im)_2_ (center). For comparison, the simulated XRD patterns of starting materials (bottom) and the product (top) are shown, as well as the intensities of selected reflections of each present phase.

The synthesis of the ZIF structures was investigated by time-resolved *in situ* XRD. In agreement with previous *in situ* studies, the parent structure ZIF-8 formation proceeds rapidly indicated by the appearance of first ZIF-8 reflections after 30 s (Batzdorf et al., 2015). The ZnO reflections gradually decrease until disappearing completely after 7 min, leaving ZIF-8 as the single product phase. Continued milling does not change the composition and crystallite size (Supplementary Figure S4). For SOD-Zn_0.9_Co_0.1_ (2Me-Im)_2_, the time-resolved *in situ* XRD data (Figure 3) follow a comparable mechanism, with slightly different detection phases of the reactant (cobalt acetate tetrahydrate visible until 30 s and zinc oxide until approximately 6 min). The data suggest that the formation of SOD-Zn_0.9_Co_0.1_ (2Me-Im)_2_ starts within the first 20 s and continues until it reaches completion after approximately 3 min.

### Zn(CF_3_-Im)_2_ by Ball Mill Grinding; Polymorphic Control by Choice of Grinding Liquid.

Fluorinated MOFs are of great interest because of their improved properties compared with their nonfluorinated counterparts. The increased hydrophobicity raises the performance in gas separation (Mondal et al., 2017; Cheplakova et al., 2018), gas storage (Zhang et al., 2013), or in the cleanup of oil spillages (Yang et al., 2011). Metal-free carbon materials with heteroatom-doping (F, N) show electrocatalytic ORR activity (Lv et al., 2017). Furthermore, with higher hydrophobicity in an ORR catalyst prepared from a fluorinated ZIF, we would expect a more efficient transport of water away from the active oxygen reduction site, resulting in improved kinetics for the ORR. Therefore, Zn(CF_3_-Im)_2_ was chosen as fluorinated analog to ZIF-8 and as a host material for Co-doping. As Zn(CF_3_-Im)_2_ can crystallize in two polymorphic crystal structures (Arhangelskis et al., 2019), the goal was to prepare both the quartz (qtz) and sodalite (SOD) topologies of the material, as well as achieving Co-doping in both of them (Schröder et al., 2013).

**Scheme 2:**
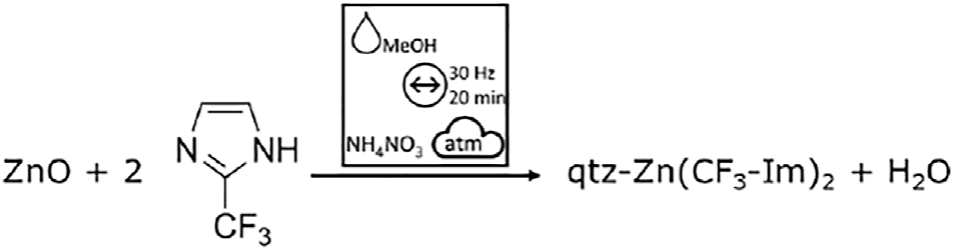
ILAG of ZnO with H-CF_3_-Im, leading to the dense qtz polymorph of Zn(CF_3_-Im)_2_.

The synthesis of the dense qtz-Zn(CF_3_-Im)_2_ polymorph was easily achieved by ILAG of zinc oxide and H-CF_3_-Im, using NH_4_NO_3_ and methanol (Scheme 2), which is in good agreement with the literature (Arhangelskis et al., 2019). The preparation of SOD-Zn(CF_3_-Im)_2_ by ILAG from zinc oxide as a starting material seems not straightforward, as SOD-Zn(CF_3_-Im)_2_ is an intermediate in the formation of qtz-Zn(CF_3_-Im)_2_. As opposed to the literature, the ethanol assisted grinding of Zn_5_(CO_3_)_2_(OH)_6_ with H-CF_3_-Im did not yield phase pure SOD-Zn(CF_3_-Im)_2_, but a mixture of the qtz and SOD polymorphs. The mechanochemical Zn(CF_3_-Im)_2_ formation by MeOH-assisted grinding of Zn_5_(CO_3_)_2_(OH)_6_ with H-CF_3_-Im was studied by time-resolved *in situ* XRD. After a short induction period (0–1 min), an interval with no detectable diffraction signals (1–5 min) is observed. From 5 min on the (100) and (101), reflections of qtz-Zn(CF_3_-Im)_2_ are detectable. The intensity of these reflections increases, and further reflections of qtz-Zn(CF_3_-Im)_2_ appear. Against our preliminary results and literature records (Arhangelskis et al., 2019), no intermediate phase of SOD-Zn(CF_3_-Im)_2_ was found. Instead, a direct conversion of starting materials into qtz-Zn(CF_3_-Im)_2_ can be observed (Supplementary Figure S5).

**FIGURE 4:**
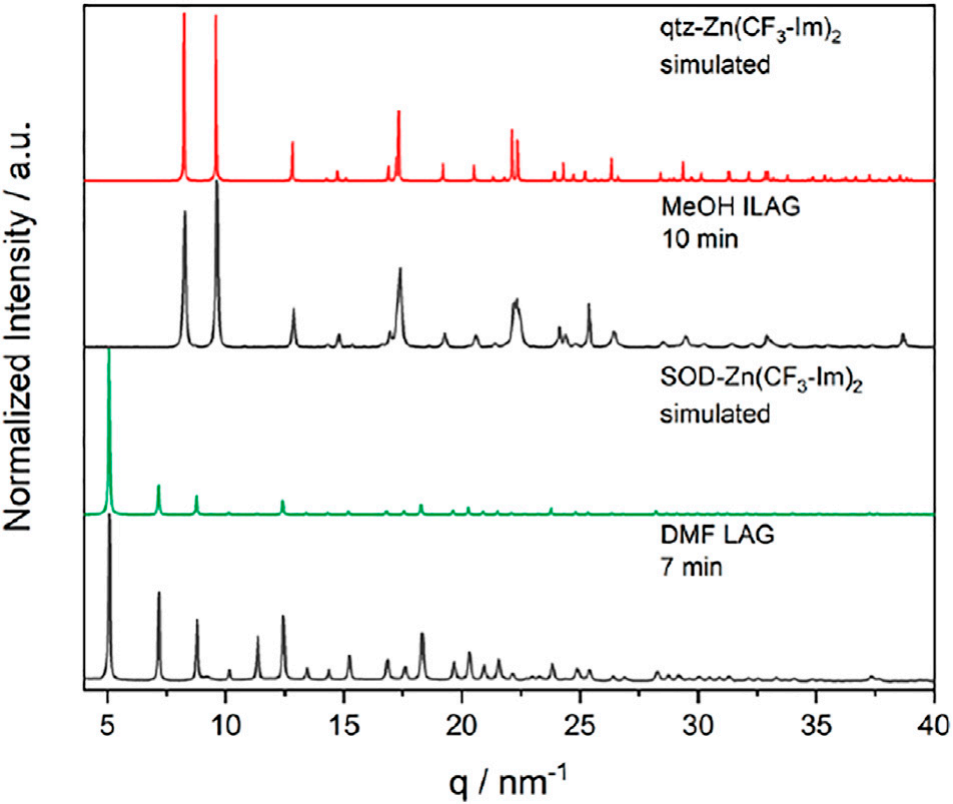
XRD of as-synthesized powders by DMF LAG and MeOH ILAG (both black) and simulated diffractograms of SOD-Zn(CF_3_-Im)_2_ (green) and qtz-Zn(CF_3_-Im)_2_ (red), indicating full conversion by ball milling.

**Scheme 3:**
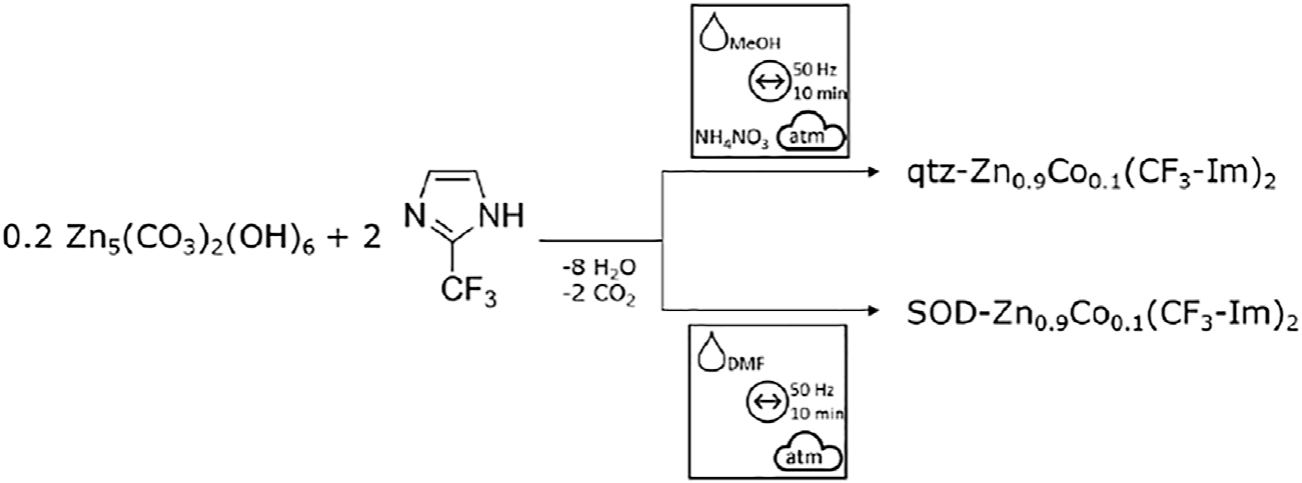
Synthesis scheme of both polymorphs of Zn(CF_3_-Im)_2_. Depending on the grinding conditions the product can be obtained as dense qtz- or porous SOD polymorph.

The *in situ* data show that under the chosen milling conditions, the reaction mechanism does not include the formation of the SOD polymorph. In a parameter study, varying milling frequency (15, 30, 50 Hz), milling ball size (3, 5, 7 mm), and added grinding liquid (MeOH, EtOH, DMF), we identified the milling conditions for the porous SOD polymorph. DMF-assisted grinding with a single 5-mm steel ball at 50 Hz yielded the SOD-Zn(CF_3_-Im)_2_, whereas MeOH ILAG leads to qtz-Zn(CF_3_-Im)_2_ (Figure 4). The milling conditions leading to both polymorphs are summarized in Scheme 3.

The mechanochemical synthesis of SOD-Zn(CF_3_-Im)_2_ was monitored by *in situ* XRD to gain insights in the formation process. The *in situ* plot shows the one-step formation of SOD-Zn(CF_3_-Im)_2_ under LAG with DMF (Supplementary Figure S6). The intensity of starting materials reflections ((200) of Zn_5_(CO_3_)_2_(OH)_6_ and (021) of H-CF_3_-Im) decreases over time, with increasing intensity of the (110) reflection of the SOD polymorph of Zn(CF_3_-Im)_2_. After approximately 6 min, the intensities of the present phases reach a plateau with little variance, correlating to the sample amount in the beam. Moreover, no conversion of the SOD polymorph into the qtz polymorph can be observed within the observed time frame.

### Co-Doping of Zn(CF_3_-Im)_2_ by Acetate ILAG/LAG Route

**Scheme 4:**
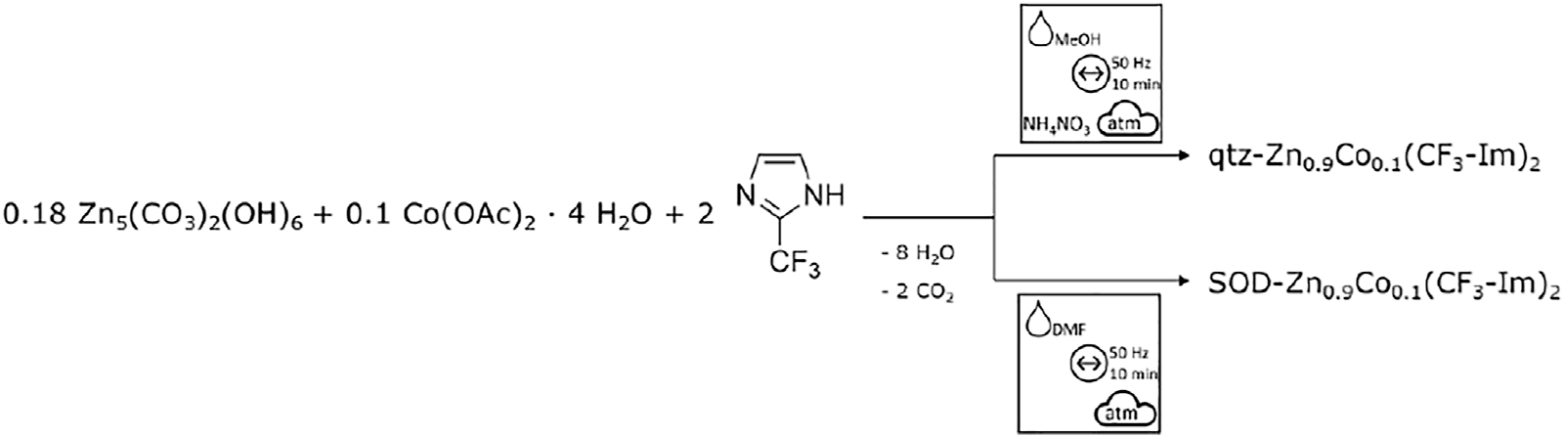
Modified synthesis route for Co-doping of both polymorphs of Zn_0.9_Co_0.1_(CF_3_-Im)_2_ by ball mill grinding.

**FIGURE 5:**
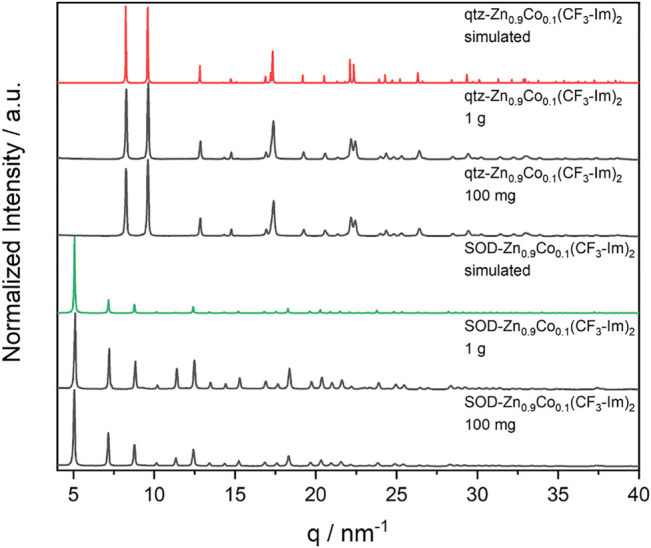
XRD data of as-synthesized powders of Zn_0.9_Co_0.1_(CF_3_-Im)_2_, in 100-mg and 1-g scale (all black) prepared by Scheme 5, with the respective goal structures SOD-Zn_0.9_Co_0.1_(CF_3_-Im)_2_ (green) and qtz-Zn_0.9_Co_0.1_(CF_3_-Im)_2_ (red).

To achieve Co-doping into the Zn(CF_3_-Im)_2_, we modified the synthesis, replacing 10 mol% of the total metal amount with cobalt acetate tetrahydrate, while keeping the milling conditions of the undoped -Zn(CF_3_-Im)_2_ (Scheme 4). Both polymorphs of Zn(CF_3_-Im)_2_ were successfully prepared by the herein presented route, in 100-mg as well as 1-g scale (Figure 5).

**FIGURE 6:**
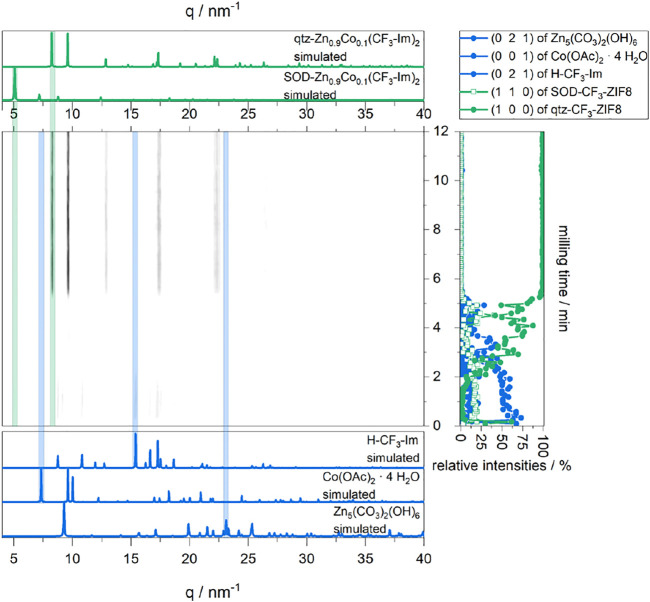
*In situ* XRD plot of the formation of qtz-Zn_0.9_Co_0.1_(CF_3_-Im)_2_ (center). For comparison, the simulated XRD patterns of starting materials (blue, bottom) and the products (green, top) are shown. The intensities of chosen reflections of the present phases are plotted on the right.

XRD was also performed for the MeOH-ILAG route to qtz-Zn_0.9_Co_0.1_(CF_3_-Im)_2_. The data in Figure 6 can be divided into several phases. In the first phase until 1 min, the intensity of starting material rises, due to more powder being in the beam. Furthermore, the (110) reflection of SOD-Zn_0.9_Co_0.1_(CF_3_-Im)_2_ appears but stays weak. Afterward, the intensity of starting materials and SOD-Zn_0.9_Co_0.1_(CF_3_-Im)_2_ decreases, until three minutes of milling time, where no crystalline phase is present any longer. From 5 min on the crystallization of qtz-Zn_0.9_Co_0.1_(CF_3_-Im)_2_ begins, visible by the rising of its (100) reflection. The single product’s maximum intensity is reached at 6.5 min, and no further changes in sample composition can be detected; thus, full conversion is reached.

**FIGURE 7:**
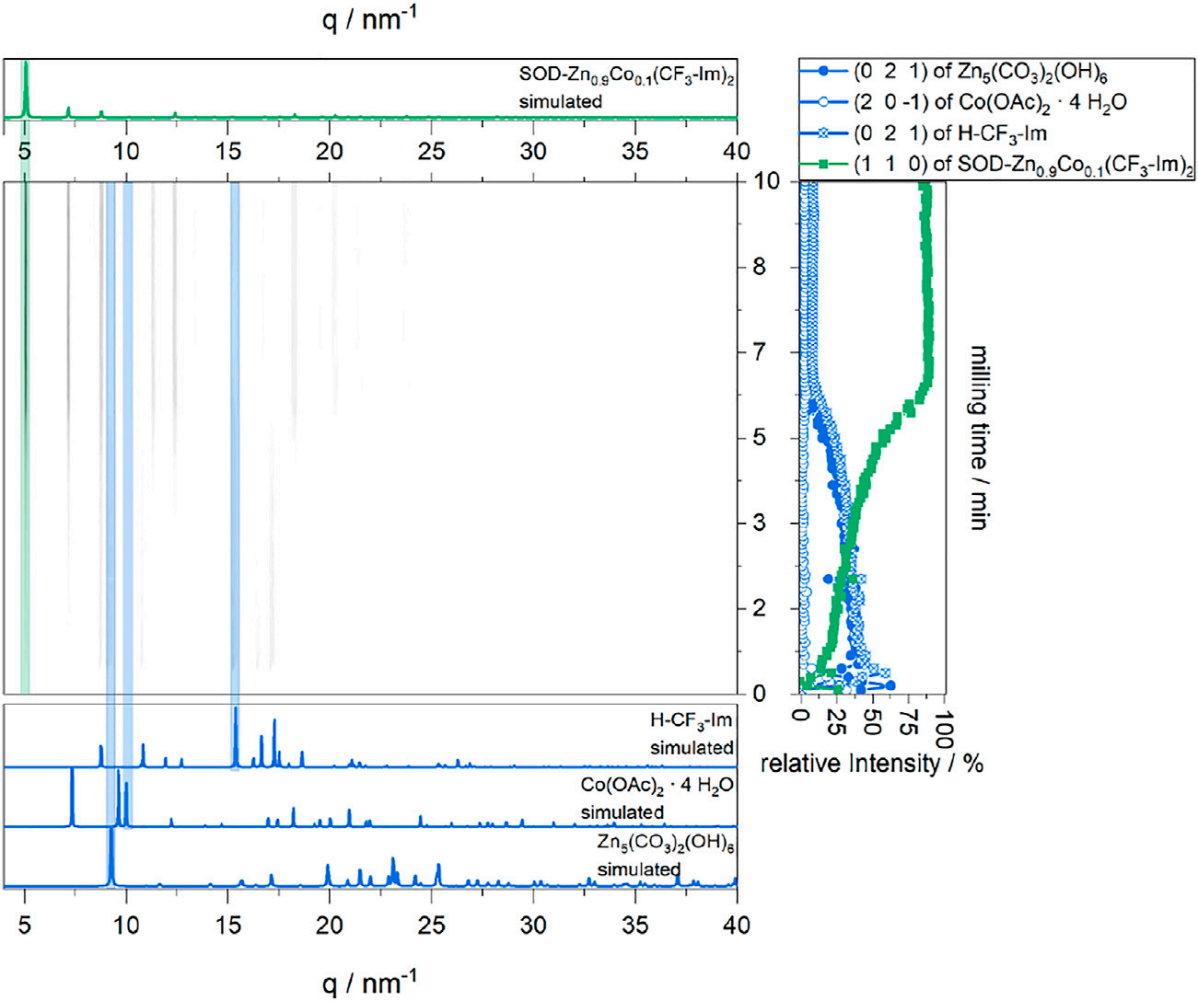
*In situ* XRD of Zn_5_(CO_3_)_2_(OH)_6_, Co(OAc)_2_ · 4 H_2_O, and H-CF_3_-Im under DMF LAG conditions, yielding (center). For comparison, the simulated XRD patterns of starting materials (blue, bottom) and the product (green, top) are shown. The intensities of chosen reflections of the present phases are plotted on the right. XPS studies on pristine and carbonized MOFs.

As the DMF LAG conditions produce the pure SOD-polymorph of Zn_0.9_Co_0.1_(CF_3_-Im)_2_ we also investigated the formation process by *in-situ* XRD. In a first phase until 30 s milling time, only the starting materials can be observed. In the second phase, their reflection intensities rise, as the milling process provides more powder into the beam. Furthermore, the (110) reflection of SOD- Zn_0.9_Co_0.1_(CF_3_-Im)_2_ appears, and its intensity rises until 1 min milling time, where it reaches a first plateau. The following phase is characterized by the gradual decrease of starting materials reflections and increase of the reflections of SOD-Zn_0.9_Co_0.1_(CF_3_-Im)_2_. After 6 min milling time, all starting materials reflections are disappeared, and after 7 min the (100) reflection of SOD- Zn_0.9_Co_0.1_(CF_3_-Im)_2_ plateaus a second time. This indicates the completion of the reaction, as no further changes, the conversion into the qtz-polymorph, can be observed (Figure 7).

**FIGURE 8:**
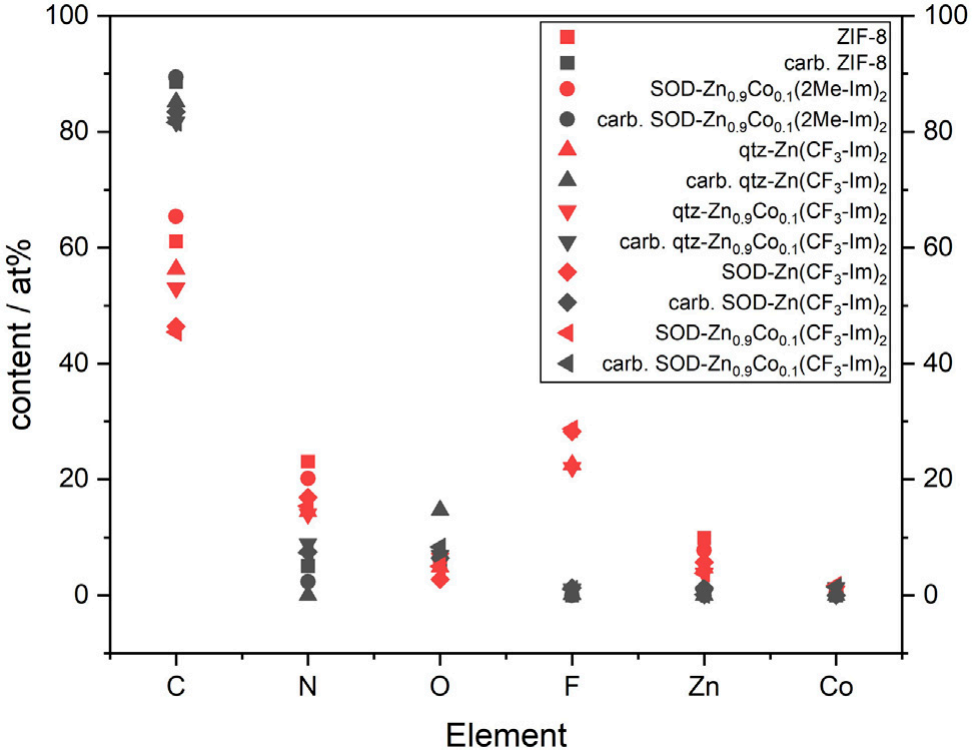
Relative elemental composition of pristine ZIFs (red) and after pyrolysis (grey). Due to decomposition the amount of N, F and Zn decrease, while C and O rise. All Co-containing samples retain Co in the same order of magnitude.

**FIGURE 9:**
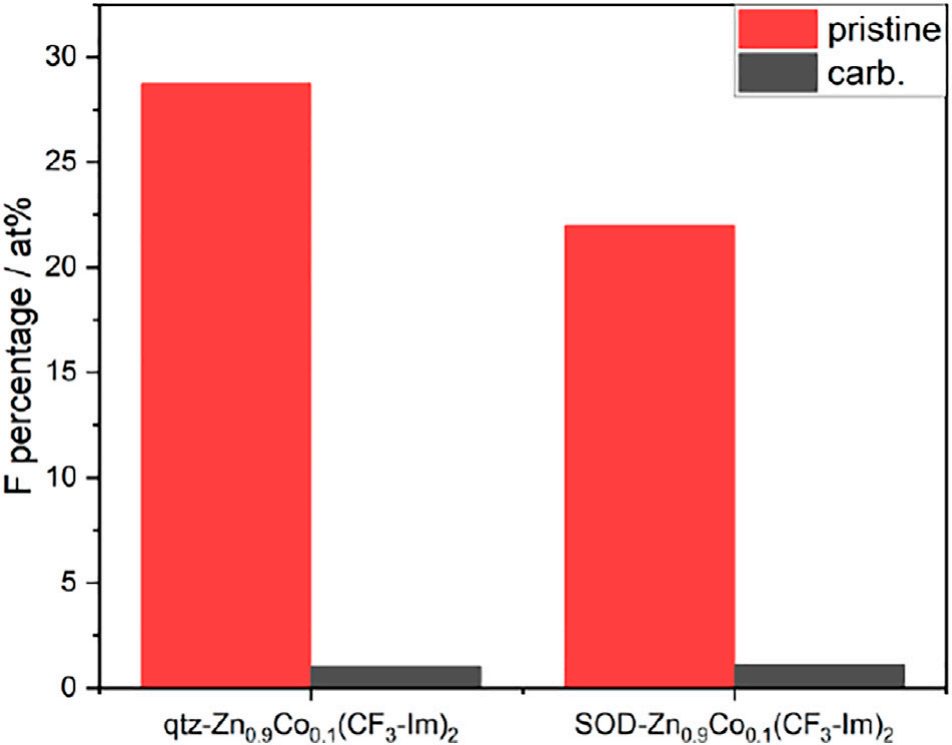
Fluorine content of pristine (red) and carbonized (grey) Zn_0.9_Co_0.1_(CF_3_-Im)_2_ in qtz- (left) and SOD- (right) topologies.

Carbonization of ZIFs is known as a method to produce nitrogen, and metal-doped carbon material (NMC) that can be applied is ORR electrocatalysis. The herein presented MOFs were therefore carbonized in a thermoscale with literature-known parameters. The samples are heated under a nitrogen atmosphere from room temperature to 900°C, where they are kept for 1 h, followed by a natural cool-down. *Ex situ* XPS was performed at the pristine MOFs and the pyrolysis products to obtain the elemental composition. As a surface-sensitive technique, XPS provides information about the outermost 10 nm of the samples. As a clear trend, it could be found that the amounts of nitrogen, fluorine, and zinc decrease, most likely due to these elements leaving by decomposition processes of the materials. As a direct consequence, the relative amount of carbon and oxygen rises. All Co-containing samples retain it in the same order of magnitude (Figure 8, 9).

The data of the fluorinated samples show for the carbonized materials the presence of two types of fluorine, metal-bound inorganic fluorine, and carbon-bound organic fluorine. In the carbonized Zn_0.9_Co_0.1_(CF_3_-Im)_2_, the organic fluorine outweighs the inorganic with a ratio of 9:1.

The high-resolution spectra of Co2p photoelectron show a Co 2p_3/2_ peak at 780.5 eV and the satellite structure typical for Co^2+^ (Biesinger et al., 2011). For Zn, the Zn 2p_3/2_ peak at 1,022 eV was observed, which can be explained with bivalent Zn (Biesinger et al., 2010). For the pyrolyzed samples, some graphitization was observed indicated by the appearance of the typical shake up peak related to the π → π * transition at 292 eV (see Supplementary Figures S9–S11).

### Electrochemical Investigations, Oxygen Reduction Reaction Catalysis

**FIGURE 10:**
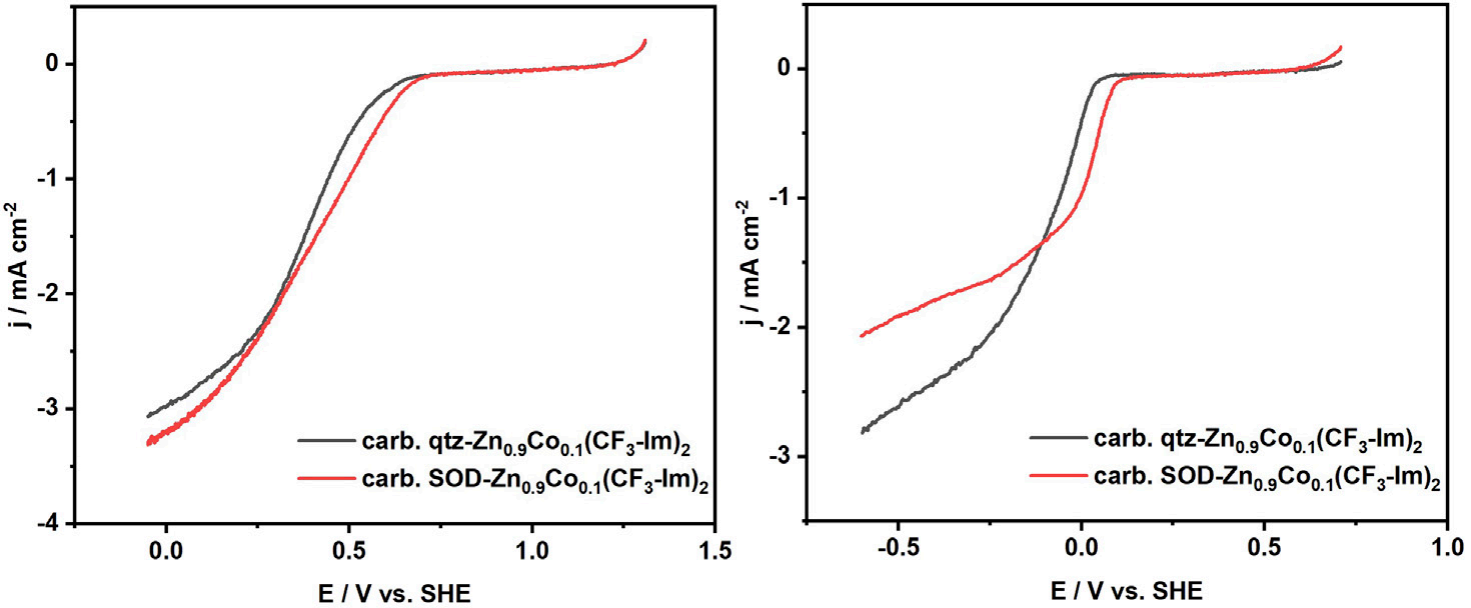
Electrochemical characterization of qtz-Zn_0.9_Co_0.1_(CF_3_-Im)_2_ and SOD-Zn_0.9_Co_0.1_(CF_3_-Im)_2_ immobilized on a RDE (1,600 rpm) by linear sweep voltammetry in O_2_-saturated **(A)** 0.1 M HClO_4_ and **(B)** 0.1 M KOH.

The performance of the ORR of pyrolyzed qtz-Zn_0.9_Co_0.1_(CF_3_-Im)_2_ and SOD-Zn_0.9_Co_0.1_(CF_3_-Im)_2_ was evaluated using the RDE. Figure 10 presents the ORR polarization curves measured in O_2_-saturated 0.1 M KOH and 0.1 M HClO_4_ electrolytes. In HClO_4_, both pyrolyzed Co-doped ZIFs exhibit a similar ORR activity with an onset potential of 0.67 V versus SHE for pyrolyzed qtz-Zn_0.9_Co_0.1_(CF_3_-Im)_2_ and a higher onset potential of 0.70 versus SHE for pyrolyzed SOD-Zn_0.9_Co_0.1_(CF_3_-Im)_2_ (Figure 10A). The half-wave potential gap between them was 22 mV, revealing a slightly higher activity of pyrolyzed SOD-Zn_0.9_Co_0.1_(CF_3_-Im)_2_. In 0.1 M KOH, the pyrolyzed SOD-Zn_0.9_Co_0.1_(CF_3_-Im)_2_ shows again a better activity toward the ORR in comparison to pyrolyzed qtz-Zn_0.9_Co_0.1_(CF_3_-Im)_2_. The onset potential of pyrolyzed SOD-Zn_0.9_Co_0.1_(CF_3_-Im)_2_ was found to be 0.12 V versus SHE with a half-wave potential of 0.0 V, whereas pyrolyzed SOD-Zn_0.9_Co_0.1_(CF_3_-Im)_2_ exhibits a lower onset potential of 0.06 V versus SHE with a half-wave potential of −0.11 V. The half-wave potential gap between both systems was 0.10 mV. Furthermore, ORR polarization curves were measured under different rotation speeds and are presented in Supplementary Figure S8. The electrocatalytic activity in O_2_-saturated electrolytes was decreasing with the decrease in rotation rate, whereas almost no activity was observed in N_2_-saturated electrolytes. Our results indicate that both materials show electrocatalytic activity for ORR; however, no significant performance improvement was evident, depending on the polymorph of Zn_0.9_Co_0.1_(CF_3_-Im)_2_ precursor.

### Summary

**FIGURE 11:**
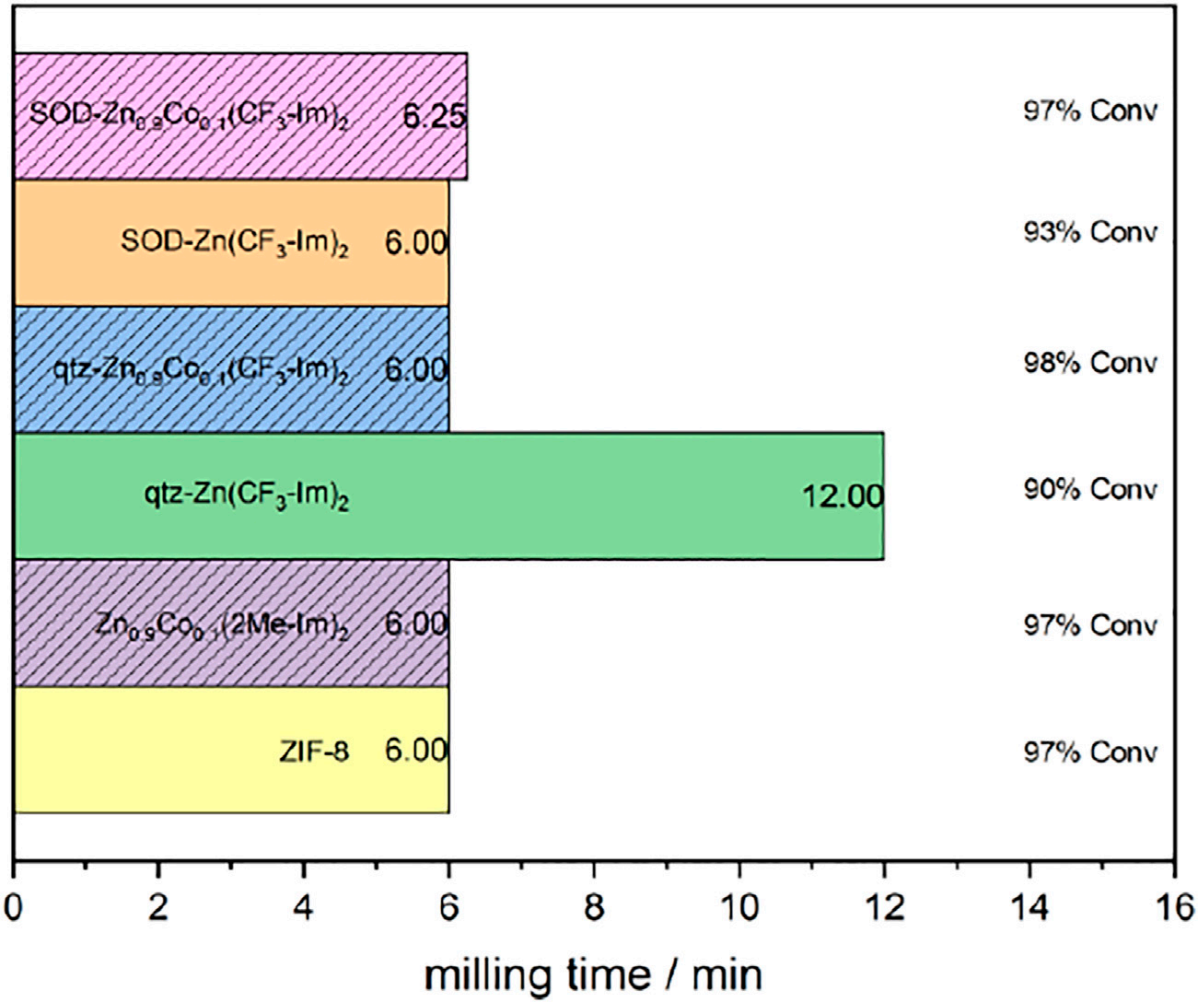
Milling times and conversion rates for the synthesis of ZIF-8, Zn_0.9_Co_0.1_ (2Me-Im)_2_ (both in SOD-topology), as well as Zn(CF_3_-Im)_2_ and Zn_0.9_Co_0.1_(CF_3_-Im)_2_ (qtz and SOD).

In this work, we present the synthesis of the first Zn_0.9_Co_0.1_(CF_3_-Im)_2_ frameworks by ball milling. Optimizing the grinding parameters allowed us to selectively produce polymorphs of Zn_0.9_Co_0.1_(CF_3_-Im)_2_. Moreover, the formation was monitored *in situ* by synchrotron XRD measurements along with the formation of ZIF-8, Zn_0.9_Co_0.1_ (2Me-Im)_2_, and Zn(CF_3_-Im)_2_ frameworks. In Figure 11, a summary of milling times and conversion rates for the synthesis of ZIF-8, Zn_0.9_Co_0.1_ (2Me-Im)_2_, and Zn(CF_3_-Im)_2_ and Zn_0.9_Co_0.1_(CF_3_-Im)_2_ (both in qtz- and SOD-topologies, respectively) is given. The data showed for all the reactions one-step transformations from starting materials into products.

Furthermore, we investigated the chemical composition after carbonization of the prepared ZIFs, finding residue fluorine, mostly of organic nature. The pyrolyzed Zn_0.9_Co_0.1_(CF_3_-Im)_2_, both in qtz and SOD topology, was successfully used as ORR electrocatalysts in acidic and alkaline media. However, no significant differences in ORR activity for both polymorphs of pyrolyzed Zn_0.9_Co_0.1_(CF_3_-Im)_2_ could be observed.

## Data Availability

The original contributions presented in the study are included in the article/Supplementary Material, further inquiries can be directed to the corresponding author.
