# Supplementary material for: Mechanochemical Synthesis of Fluorine-Containing Co-Doped Zeolitic Imidazolate Frameworks for Producing Electrocatalysts
Source: Front Chem. 2022 Mar 14;10:840758. doi: 10.3389/fchem.2022.840758 (PMC8964432; doi:10.3389/fchem.2022.840758)
Supplement: Supplementary file 1 [file DataSheet1.docx]

Supplementary Material

Mechanochemical synthesis of fluorine-containing Co-doped zeolitic imidazolate frameworks for producing electrocatalysts

Max Rautenberg,^1,2^ Marius Gernhard,^3^ Jörg Radnik,^1^ Julia Witt,^1^ Christina Roth,^3^ Franziska Emmerling^1,2*^

^1^BAM Federal Institute of Materials Research and Testing, Richard-Willstätter-Str. 11, 12489 Berlin, Germany

^2^Humboldt-Universität zu Berlin, Department of Chemistry, Brook-Taylor-Str. 2, 12489 Berlin, Germany

^3^ Universität Bayreuth, Universitätsstr. 30, 95447 Bayreuth, Germany


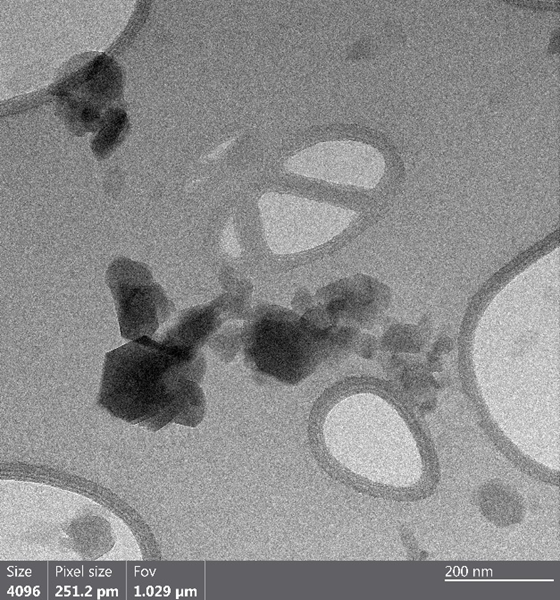


**Supplementary Figure 1:** TEM image of Zn_0.9_Co_0.1_(2Me-Im)_2_ after mechanochemical synthesis. Next to highly geometric particles, particles with an uneven size distribution and shape are visible.


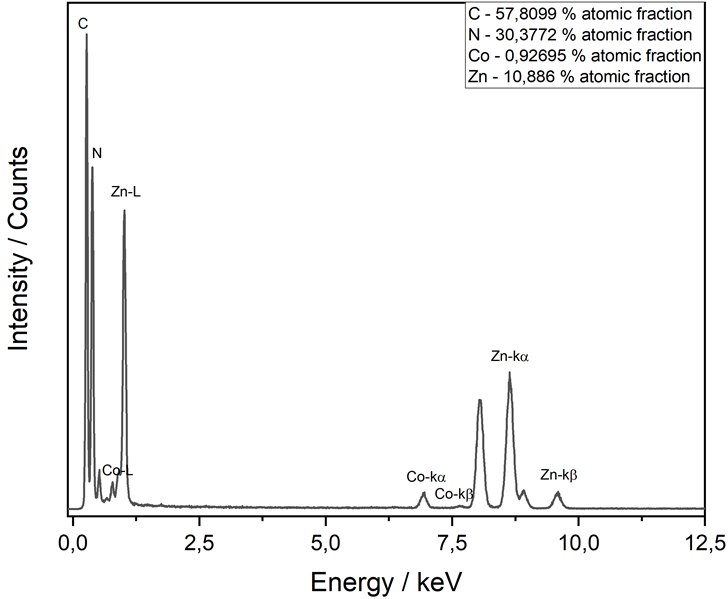


Supplementary Figure 2: Analysis of the Zn_0.9_Co_0.1_(2Me-Im)_2_ by energy dispersive X-ray spectroscopy (EDS) revealing the a Co/(Zn+Co) ration of 7.85, close to the expected 10 mol%. This confirms the successfull doping of cobalt into the ZIF-8 structure by the acetate ILAG route.


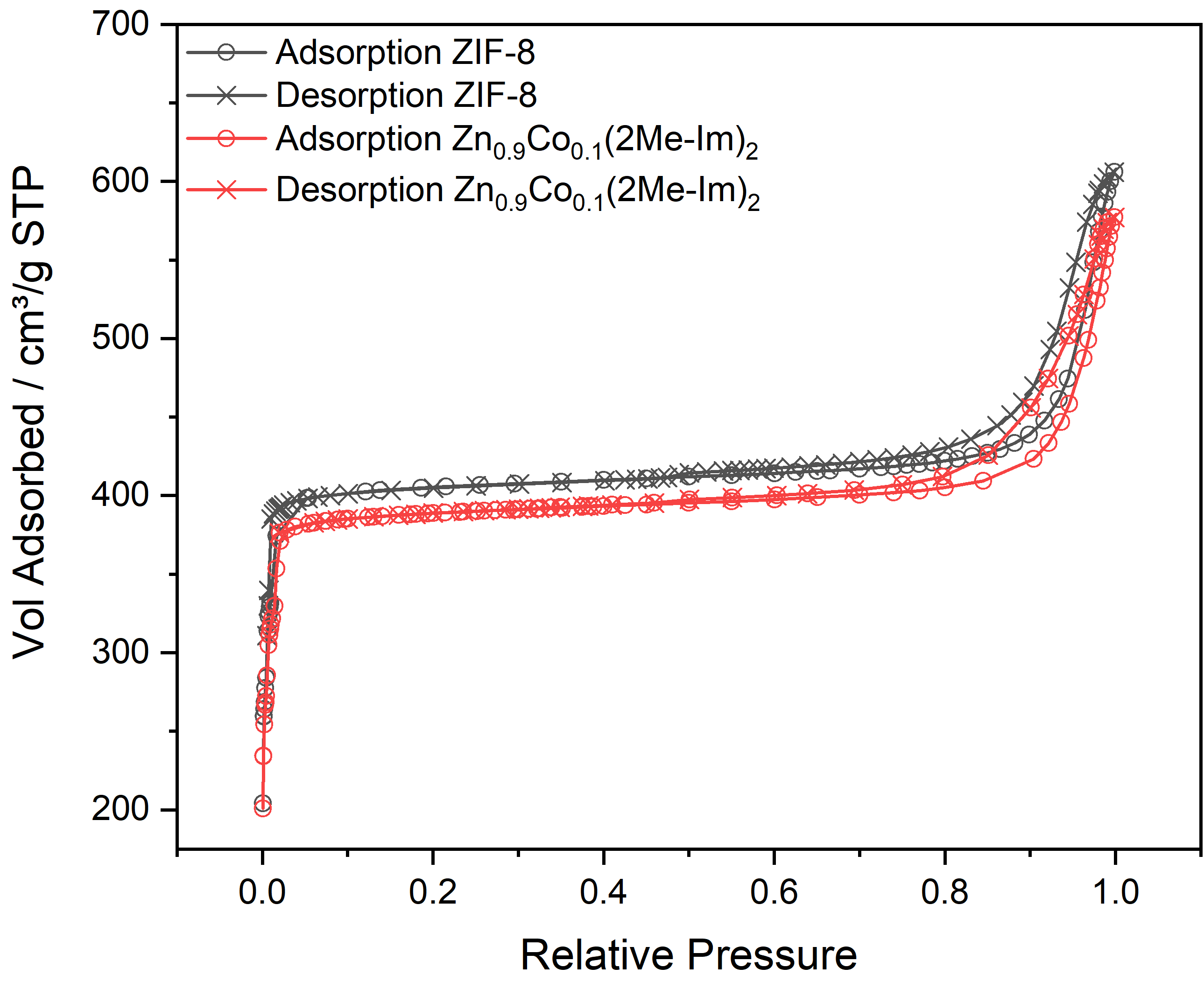


Supplementary Figure 3: Nitrogen ad- and desorption Isotherms at 77 K of mechanochemically prepared ZIF-8 and Zn_0.9_Co_0.1_(2Me-Im)_2_ after activation in methanol.


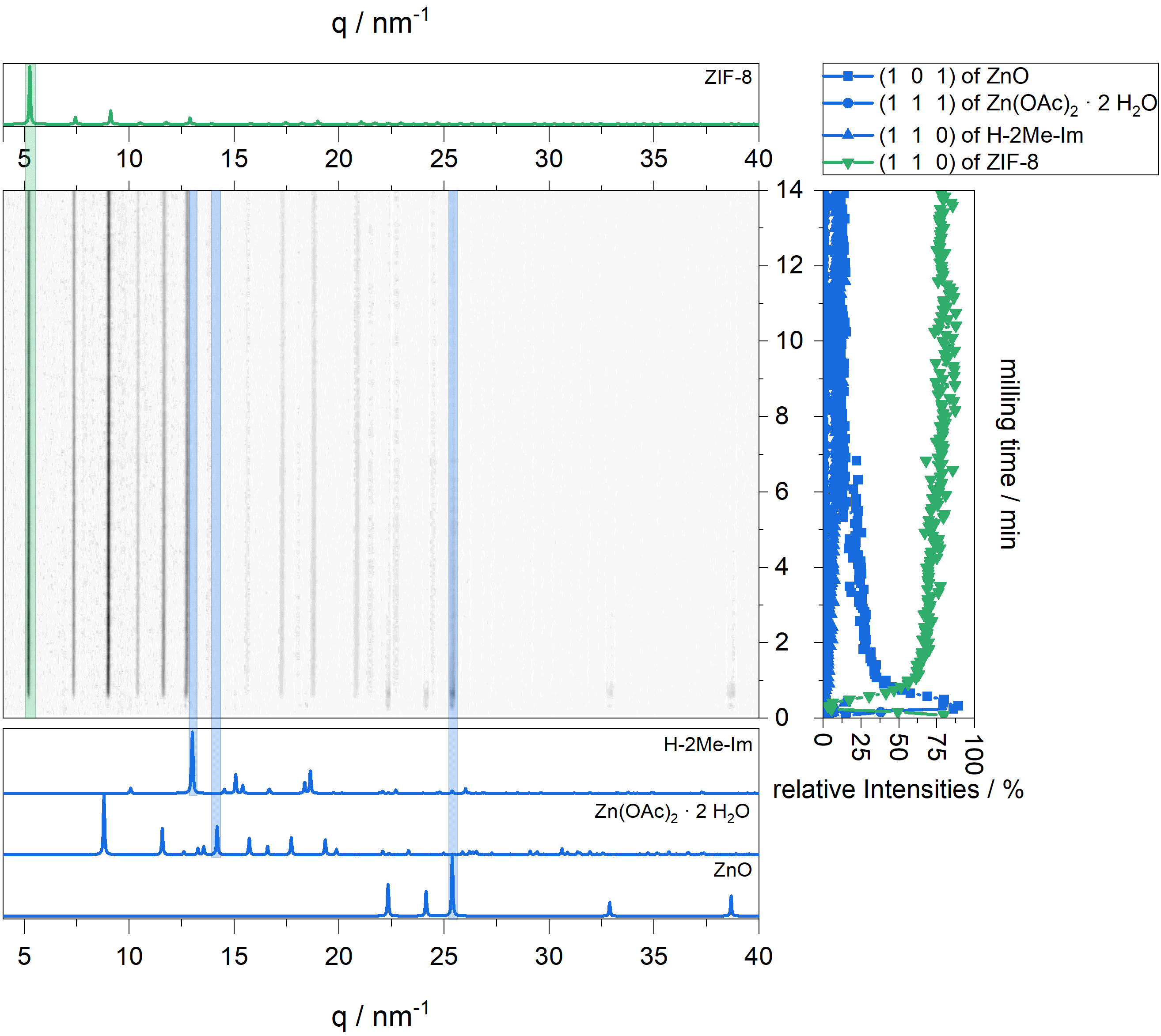


Supplementary Figure 4: In situ PXRD plot of the formation of ZIF-8 (center). For comparison the simulated XRD patterns of starting materials (bottom) and the product (top) are shown, as well as the intensities of selected reflections of each present phase.


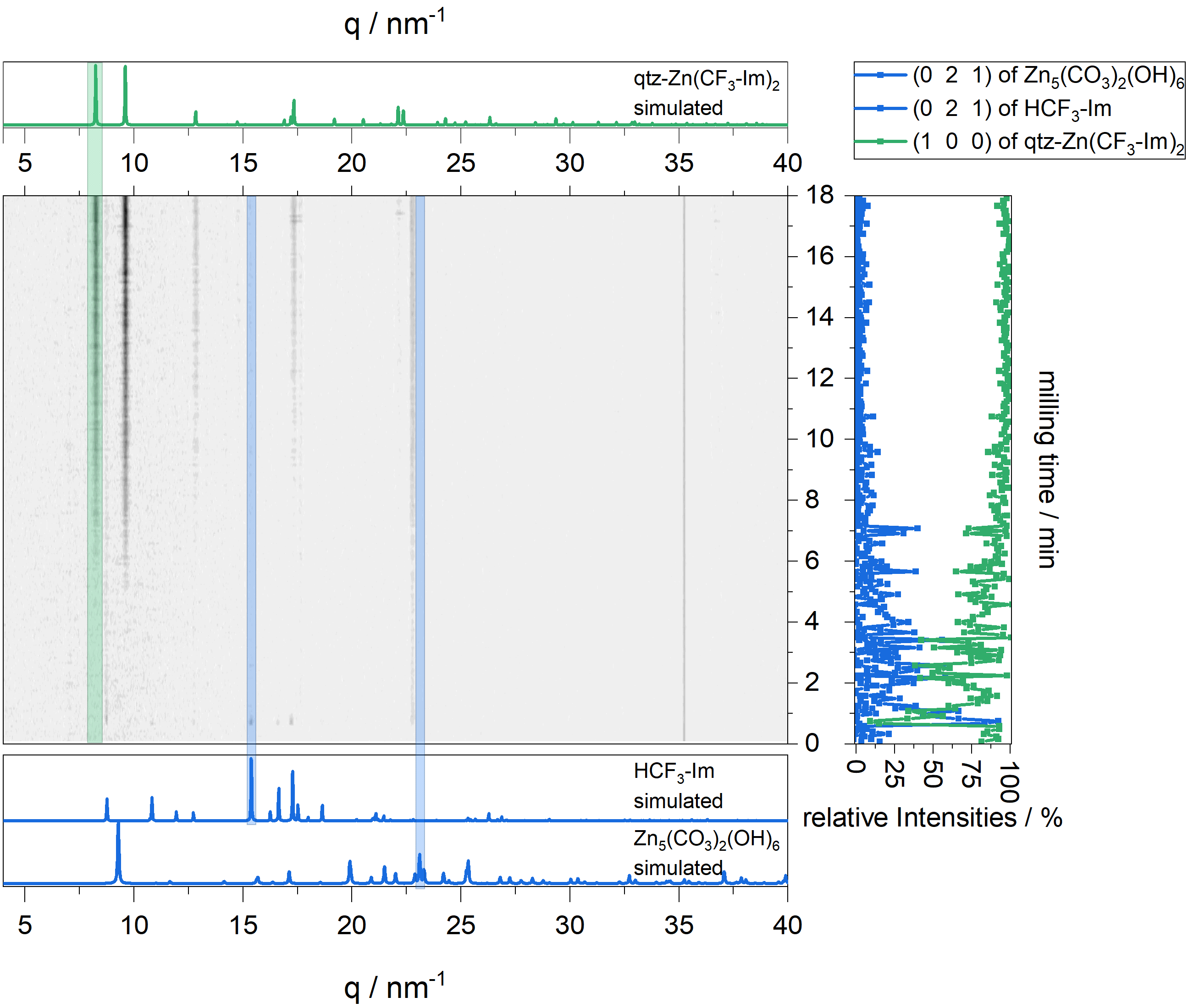


Supplementary Figure 5: In situ XRD plot of the formation of qtz-Zn(CF_3_-Im)_2_ (center) under MeOH assisted grinding. For comparison the simulated XRD patterns of starting materials (bottom) and the product (top) are shown. Additionally, relative intensities of present phases, based on selected reflections (green: (100) of qtz-Zn(CF_3_-Im)_2_, blue: (021) of HCF_3_-Im and (021) of Zn_5_(CO_3_)_2_(OH)_6_) are depicted on the right.


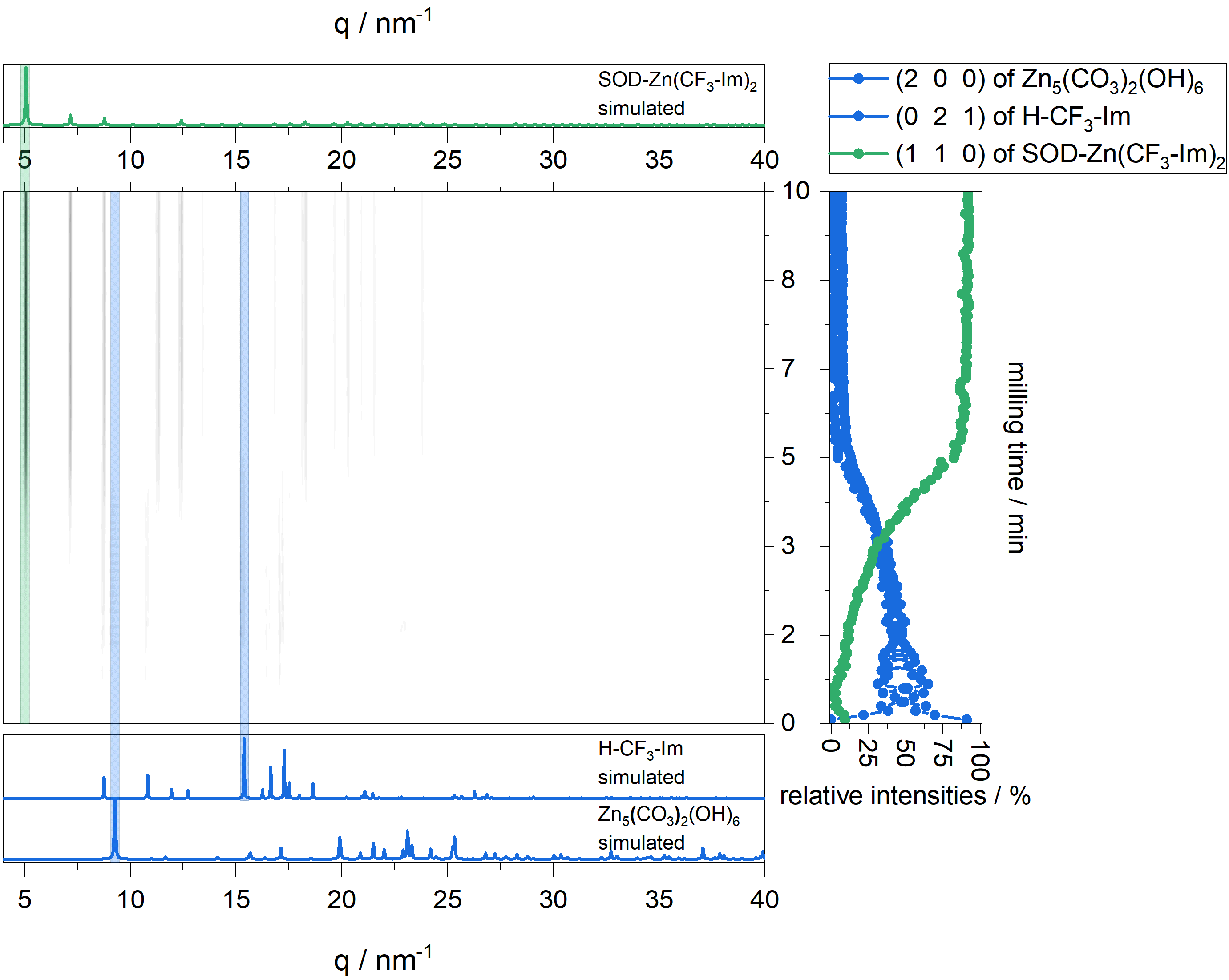


Supplementary Figure 6: In situ XRD plot of the formation of SOD-Zn(CF_3_-Im)_2_ (center) under DMF-assisted grinding. For comparison the simulated XRD patterns of starting materials (bottom) and the product (top) are shown, as well as the intensities of chosen reflections (green: (110) of SOD-Zn(CF_3_-Im)_2_, blue: (021) of H-CF_3_-Im and (200) of Zn_5_(CO_3_)_2_(OH)_6_) during the milling.


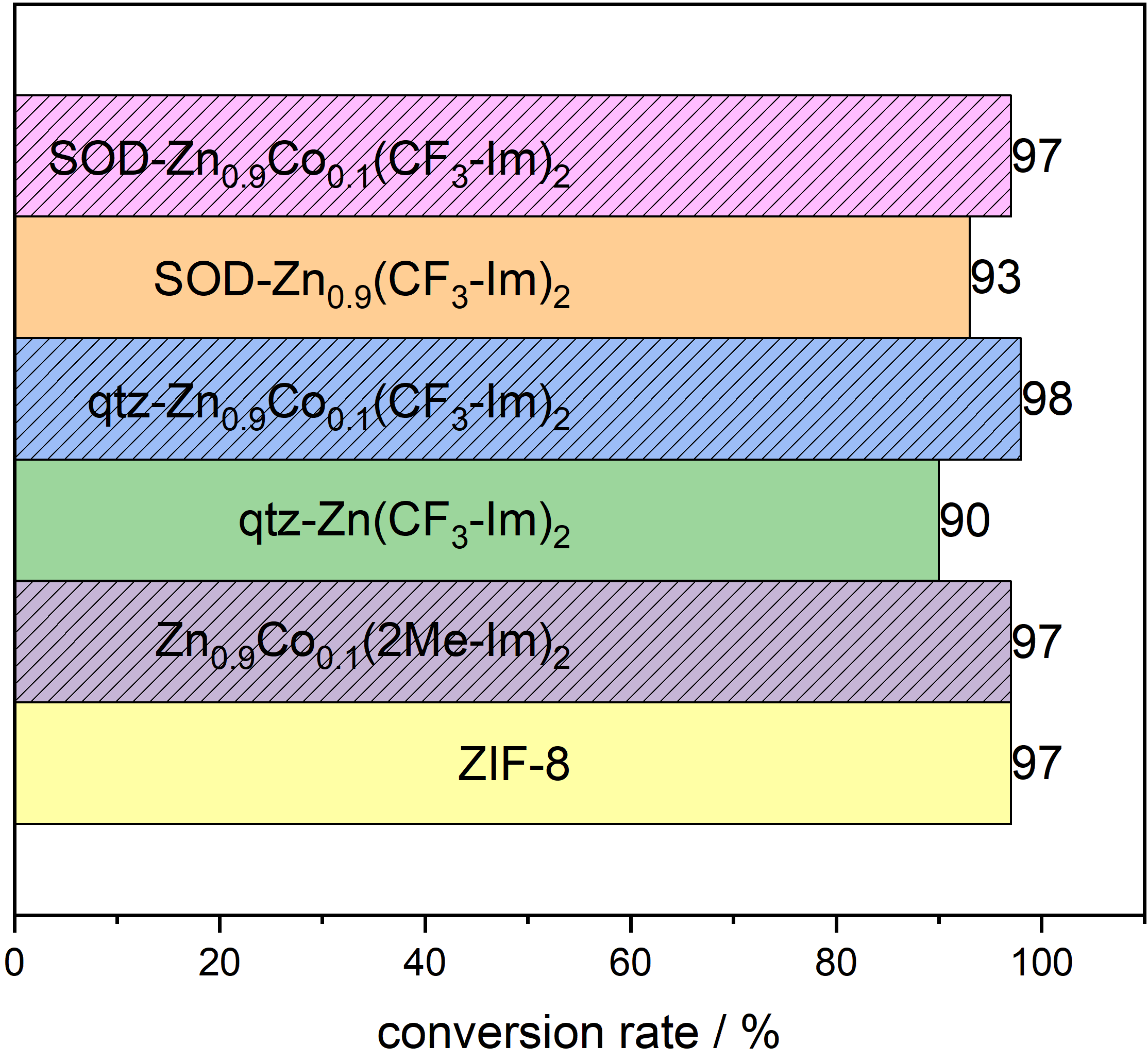


Supplementary Figure 7: Conversion rates after 15 min milling time for the synthesis of ZIF-8, Zn_0.9_Co_0.1_(2Me-Im)_2_, Zn(CF_3_-Im)_2_ and Zn_0.9_Co_0.1_(CF_3_-Im)_2_ (both qtz- and SOD-topology).


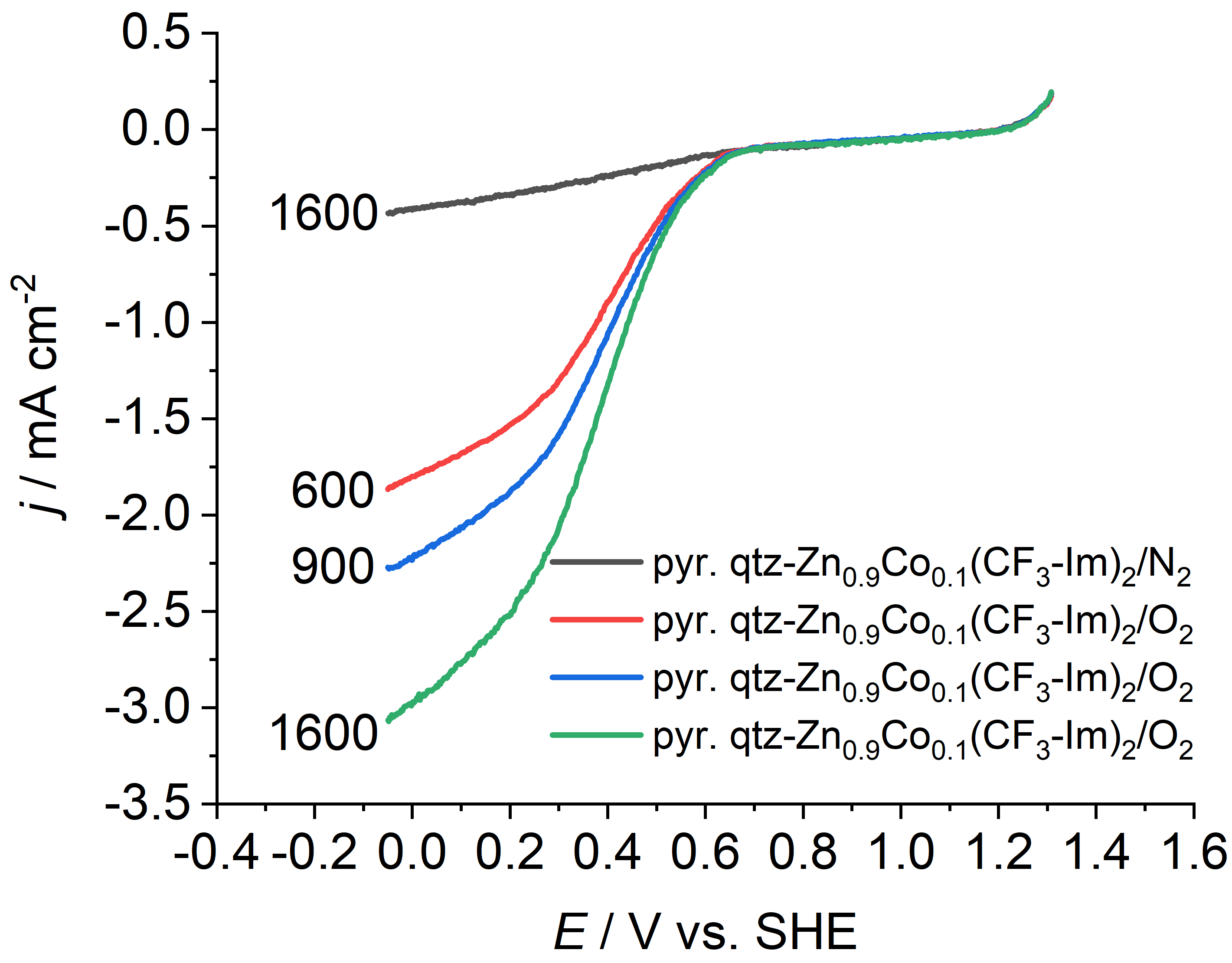

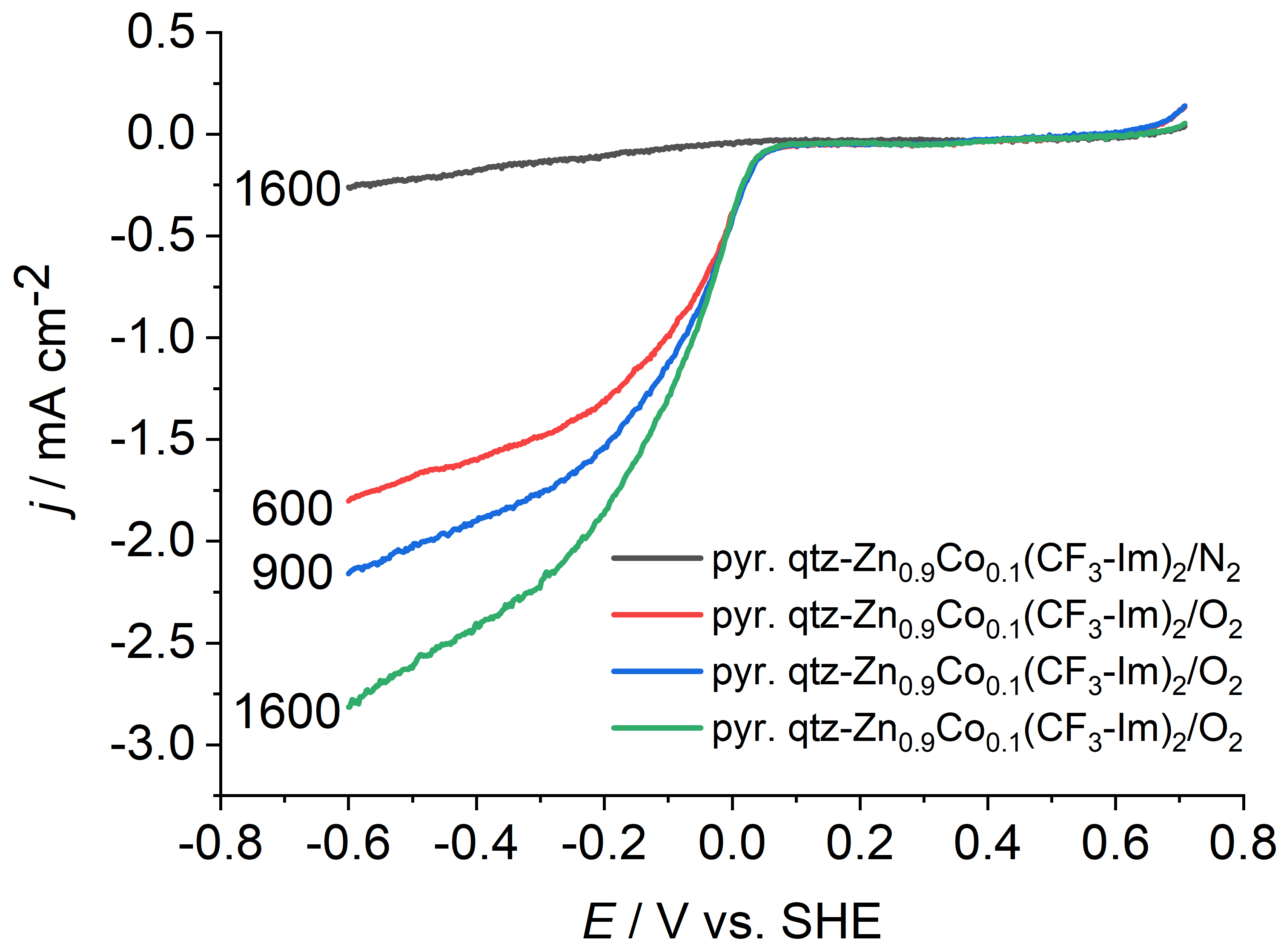

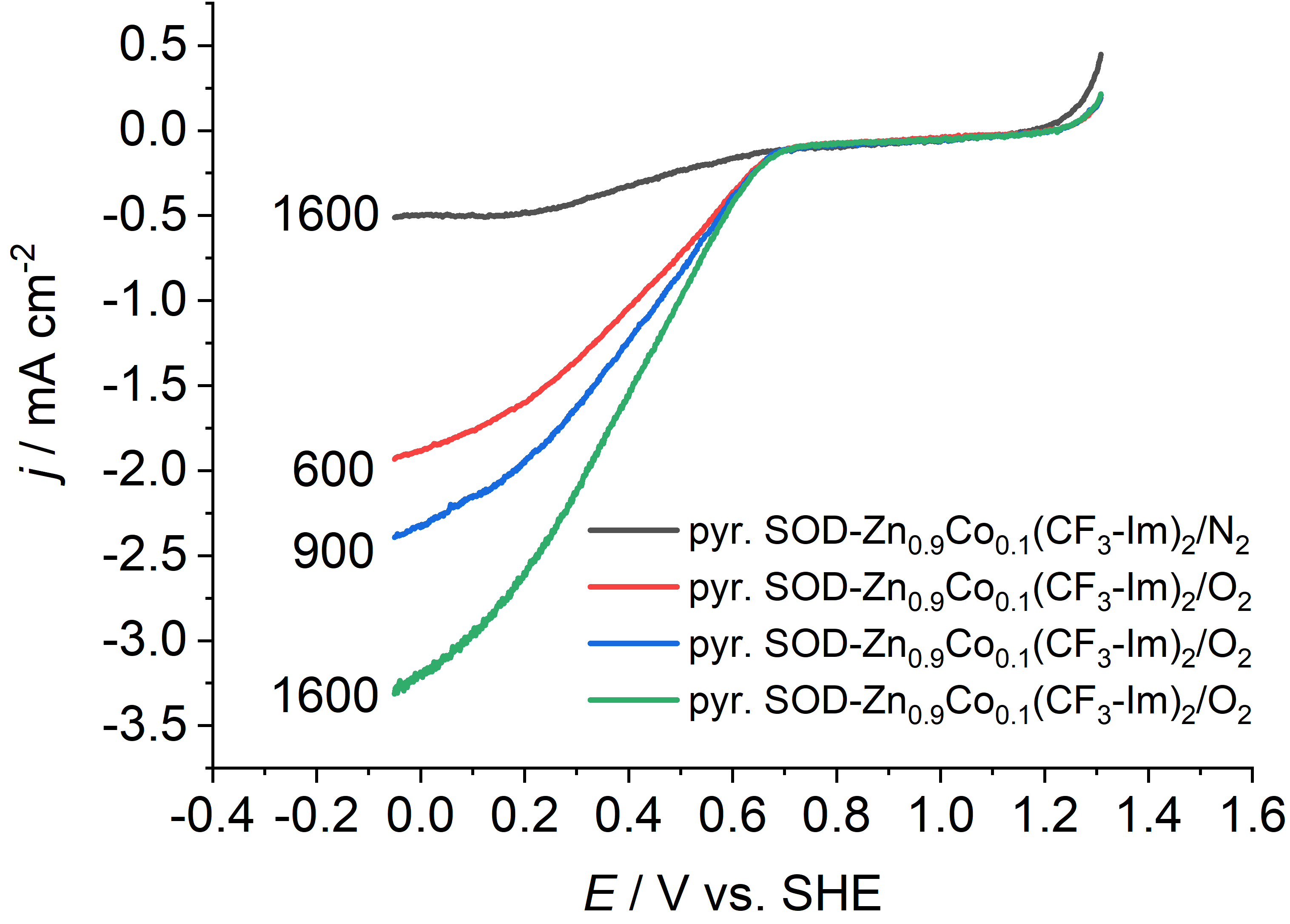

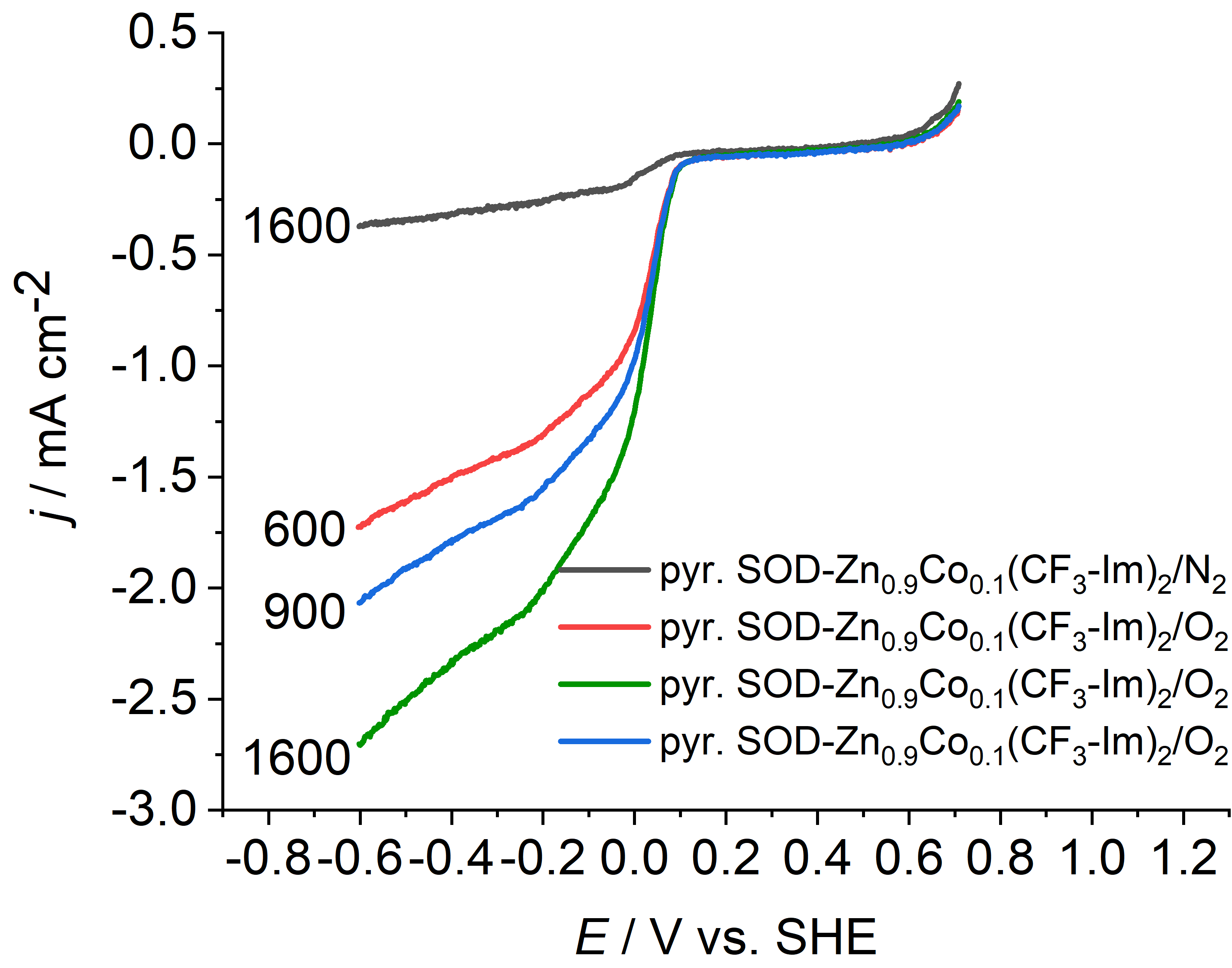


c)

d)

b)

a)

**Supplementary Figure 8:** ORR polarization curves of a-b) qtz-Zn_0.9_Co_0.1_(CF_3_-Im)_2_ and c-d) SOD-Zn_0.9_Co_0.1_(CF_3_-Im)_2_ measured in N_2_- and O_2_-saturated 0.1 M HClO_4_ and 0.1 M KOH at different rotation rates of 600, 900 and 1600 rpm at a scan rate of 20 mV^s-1^.


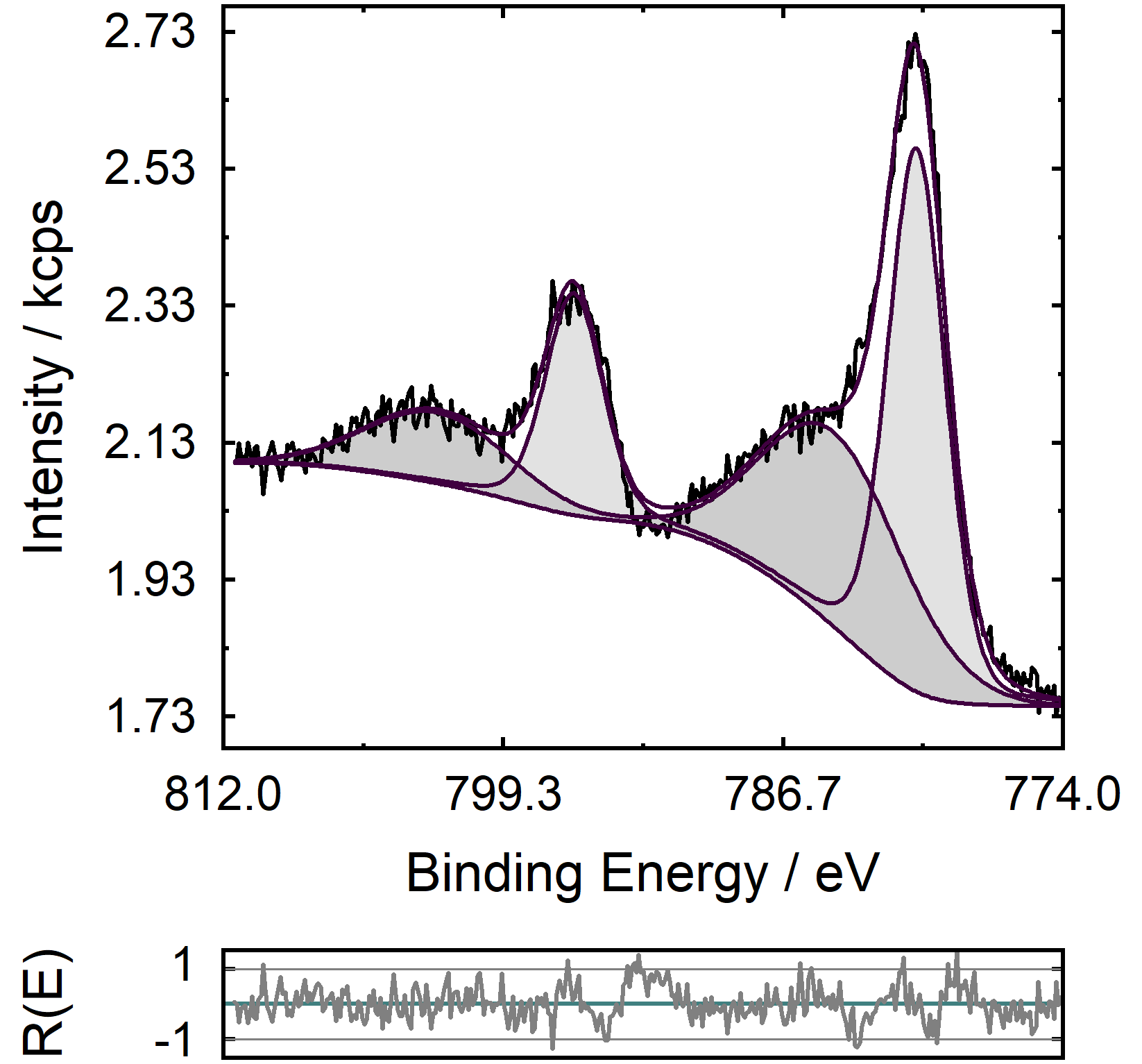


**Supplementary Figure 9:** Fit of the Co 2p high-resolution spectrum of carb. SOD-Zn_0.9_Co_0.1_(CF_3_-Im)_2_.

**Supplementary Table 1:** Fitting data for the Co 2p signal in carb. SOD-Zn_0.9_Co_0.1_(CF_3_-Im)_2_

| **doublet name Co 2p** | **peak height / cps** | **lorentzian** | **position / eV** | **FWHM / eV** | **abs. area** | **rel. area / %** |
| --- | --- | --- | --- | --- | --- | --- |
| **Co 2p main** | **793**  **323.06** | **0.2**  **0.2** | **780.8359**  **796.3424** | **3.0658**  **3.0658** | **2767**  **1138** | **39.22**  **16.13** |
| **Co 2p sat.** | **283.713**  **103.505** | **0**  **0** | **784.5397**  **802.5443** | **7.6504**  **7.6504** | **2309**  **841** | **32.73**  **11.92** |


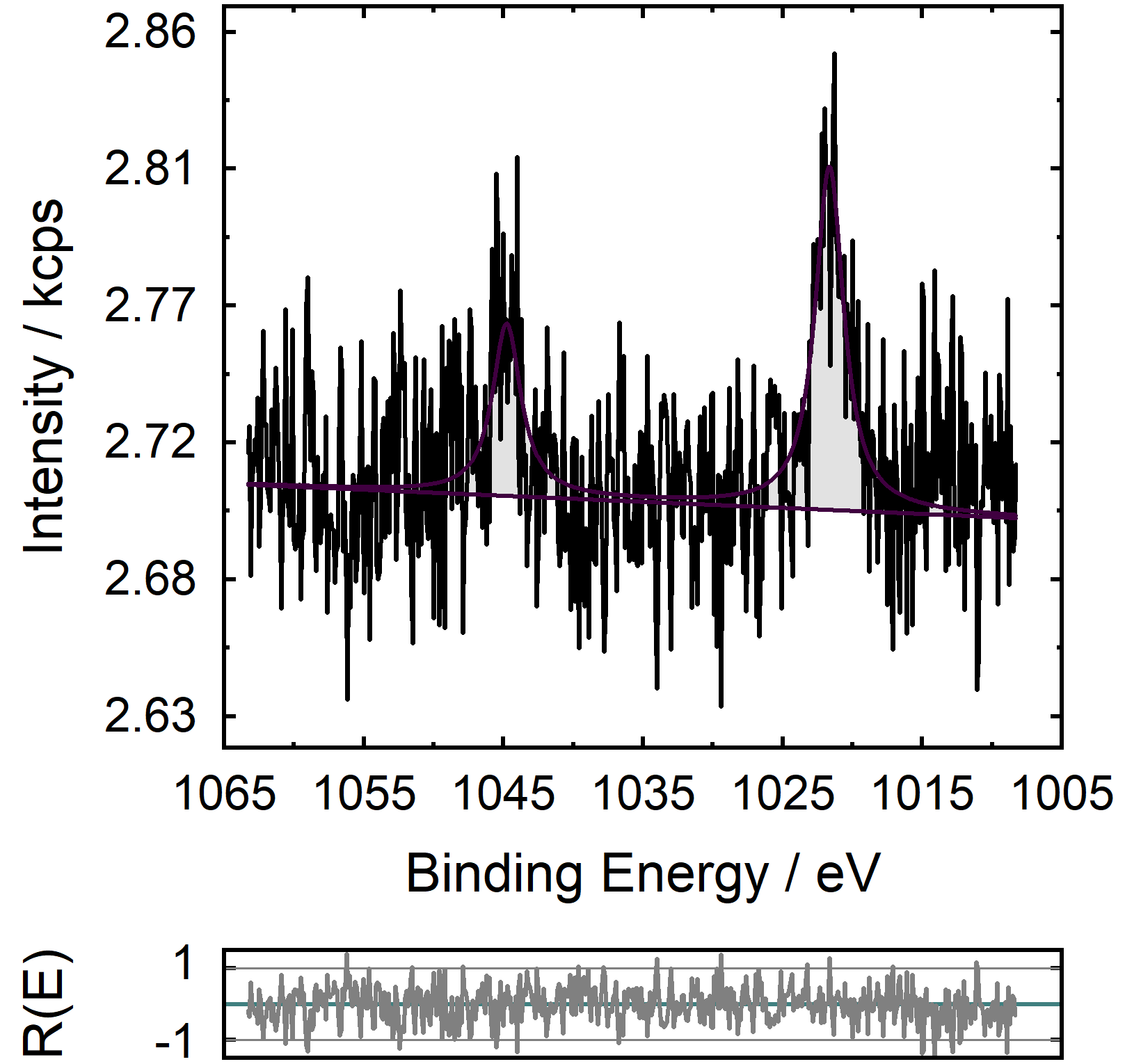


**Supplementary Figure 10:** Fit of the Zn 2p high-resolution spectrum of carb. SOD-Zn_0.9_Co_0.1_(CF_3_-Im)_2_.

**Supplementary Table 2:** Fitting data for the Zn 2p signal in carb. SOD-Zn_0.9_Co_0.1_(CF_3_-Im)_2_

| **doublet name Zn 2P** | **peak height / cps** | | **lorentzian** | **position / eV** | **FWHM / eV** | **abs. area** | **rel. area / %** |
| --- | --- | --- | --- | --- | --- | --- | --- |
| **Zn 2p** | **115.436**  **57.718** | **0.96337**  **0.96337** | | **1021.862**  **1044.962** | **2.47226**  **2.47226** | **426.28**  **214.61** | **66.51**  **33.49** |


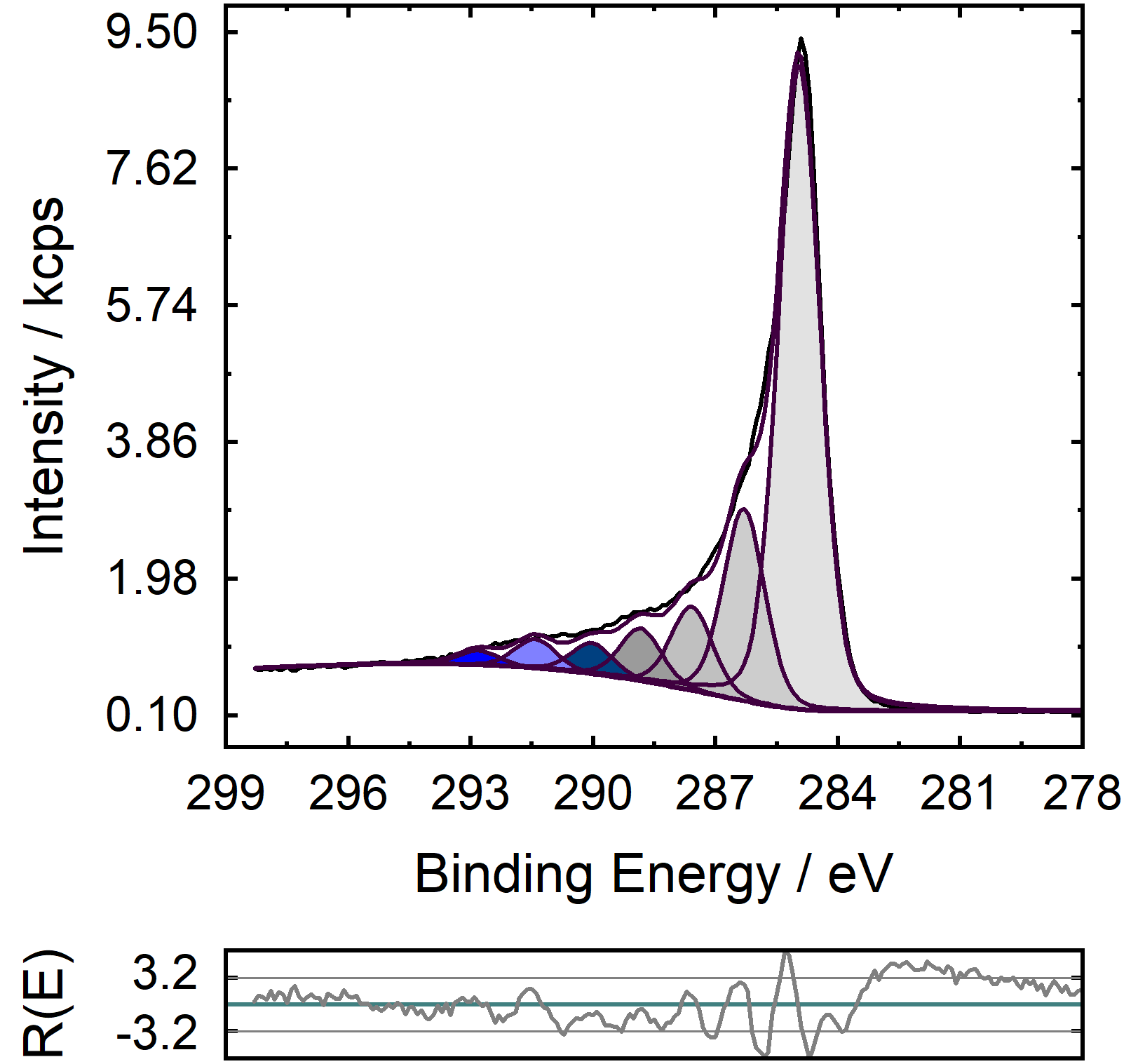


**Supplementary Figure 11:** Fit of the C 1s high-resolution spectrum of carb. SOD-Zn_0.9_Co_0.1_(CF_3_-Im)_2_.

ZIF-8

**Supplementary Figure 12:** XPS spectrum of ZIF-8.

Supplementary Table 3: Analysis data for ZIF-8

| **peak name** | **E_B_ / eV** | **area /cps eV** | **sens fact.** | **norm. area** | **quant. / at. %** |
| --- | --- | --- | --- | --- | --- |
| **C 1s** | **283.5** | **117670.6** | **18775** | **6.267114** | **61.09** |
| **N 1s** | **396.5** | **82681.52** | **34926** | **2.367299** | **23.08** |
| **O 1s** | **529.5** | **34848.87** | **57111** | **0.6101952** | **5.95** |
| **Zn 2p** | **1019.5** | **335081.3** | **330412** | **1.014131** | **9.89** |

pyrolyzed ZIF-8

Supplementary Figure 13: XPS spectrum of pyrolyzed ZIF-8.

Supplementary Table 4: Analysis data for pyrolyzed ZIF-8

| **peak name** | **E_B_ / eV** | **area /cps eV** | **sens fact.** | **norm. area** | **quant. / at. %** |
| --- | --- | --- | --- | --- | --- |
| **C 1s** | **282.5** | **262551.9** | **18766** | **13.99063** | **88.6** |
| **N 1s** | **396.5** | **27765.08** | **34926** | **0.7949568** | **5.03** |
| **O 1s** | **529.5** | **51593.05** | **57111** | **0.9033817** | **5.72** |
| **Zn 2p** | **1018.5** | **33814.07** | **331028** | **0.1021486** | **0.65** |

SOD-Zn_0.9_Co_0.1_(2Me-Im)_2_

**Supplementary Figure 14:** XPS spectrum of SOD-Zn_0.9_Co_0.1_(2Me-Im)_2_.

**Supplementary Table 5:** Analysis data for SOD-Zn_0.9_Co_0.1_(2Me-Im)_2_

| **peak name** | **E_B_ / eV** | **area /cps eV** | **sens fact.** | **norm. area** | **quant. / at. %** |
| --- | --- | --- | --- | --- | --- |
| **C 1s** | **285.5** | **118584.8** | **18785** | **6.312474** | **65.4** |
| **N 1s** | **399.5** | **68012.66** | **34943** | **1.946374** | **20.17** |
| **O 1s** | **531.5** | **33896.81** | **57084** | **0.5938036** | **6.15** |
| **Co 2p** | **779.5** | **15623.33** | **326924** | **0.0477887** | **0.5** |
| **Zn 2p** | **1019.5** | **248384.6** | **330412** | **0.7517415** | **7.79** |


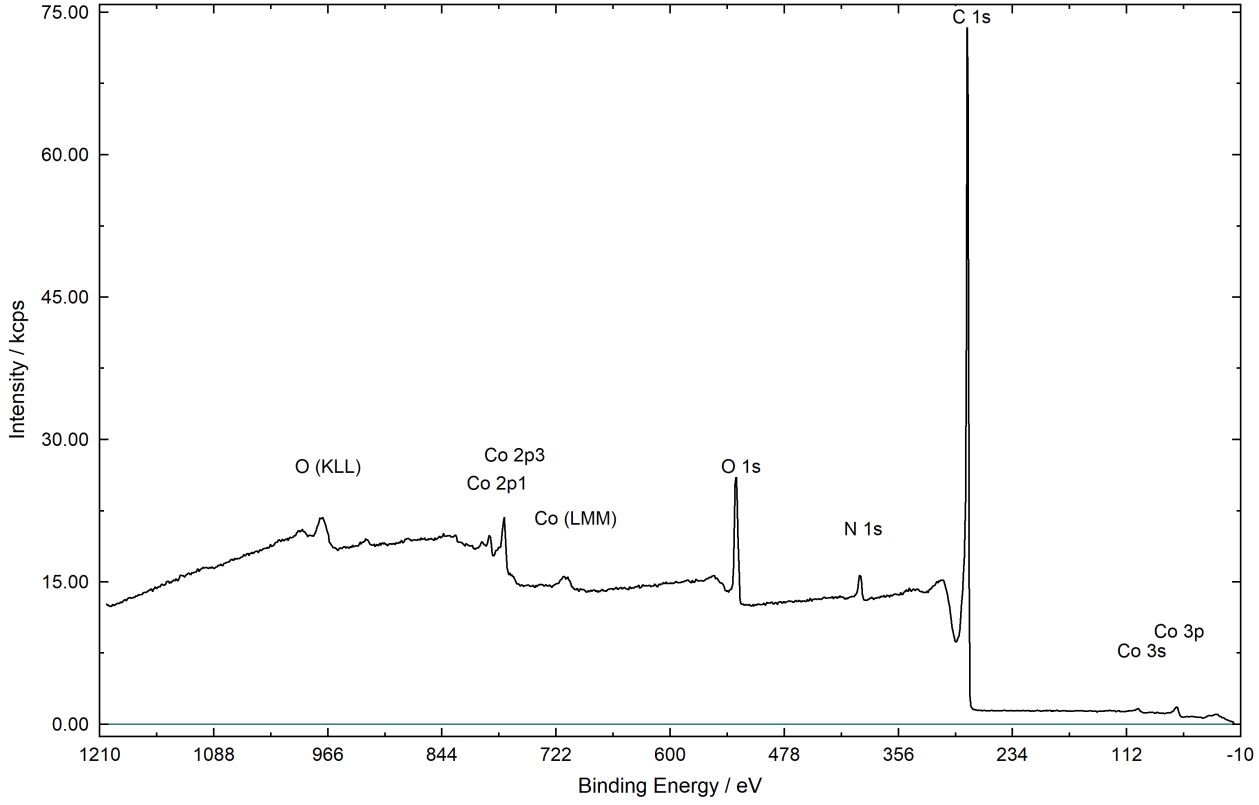


pyrolyzed SOD-Zn_0.9_Co_0.1_(2Me-Im)_2_

**Supplementary Figure 15:** XPS spectrum of pyrolyzed SOD-Zn_0.9_Co_0.1_(2Me-Im)_2_.

**Supplementary Table 6:** Analysis data of pyrolyzed SOD-Zn_0.9_Co_0.1_(2Me-Im)_2_

| **peak name** | **E_B_ / eV** | **area /cps eV** | **sens fact.** | **norm. area** | **quant. / at. %** |
| --- | --- | --- | --- | --- | --- |
| **C 1s** | **282.5** | **252244** | **18766** | **13.44135** | **89.41** |
| **N 1s** | **397.5** | **12461.33** | **34938** | **0.3566661** | **2.37** |
| **O 1s** | **529.5** | **59966.44** | **57111** | **1.049998** | **6.98** |
| **Co 2p** | **777.5** | **600926.3** | **327602** | **0.1859764** | **1.24** |

qtz-Zn(CF_3_-Im)_2_

**Supplementary Figure 16:** XPS spectrum of qtz-Zn(CF_3_-Im)_2_.

**Supplementary Table 7:** Analysis data of qtz-Zn(CF_3_-Im)_2_

| **peak name** | **E_B_ / eV** | **area /cps eV** | **sens fact.** | **norm. area** | **quant. / at. %** |
| --- | --- | --- | --- | --- | --- |
| **C 1s** | **283.5** | **108200.9** | **18775** | **5.762758** | **53.36** |
| **N 1s** | **397.5** | **54433.18** | **34938** | **1.557977** | **14.43** |
| **O 1s** | **530.5** | **30500.39** | **57080** | **0.5343353** | **4.95** |
| **F 1s** | **685.5** | **198448.8** | **81508** | **2.434689** | **22.54** |
| **Zn 2p** | **1020.5** | **168128.6** | **329466** | **0.5103059** | **4.73** |

pyrolyzed qtz-Zn(CF_3_-Im)_2_

**Supplementary Figure 17:** XPS spectrum of pyrolyzed qtz-Zn(CF_3_-Im)_2_.

**Supplementary Table 8:** Analysis data of pyrolyzed qtz-Zn(CF_3_-Im)_2_

| **peak name** | **E_B_ / eV** | **area /cps eV** | **sens fact.** | **norm. area** | **quant. / at. %** |
| --- | --- | --- | --- | --- | --- |
| **C 1s** | **282.5** | **238576.7** | **18766** | **12.71306** | **85.14** |
| **O 1s** | **530.5** | **125546.1** | **57080** | **2.199439** | **14.73** |
| **F 1s** | **685.5** | **1641.997** | **81508** | **0.020145** | **0.13** |

qtz-Zn_0.9_Co_0.1_(CF_3_-Im)_2_

**Supplementary Figure 18:** XPS spectrum of qtz-Zn_0.9_Co_0.1_(CF_3_-Im)_2_.

**Supplementary Table 9:** Analysis data of qtz-Zn_0.9_Co_0.1_(CF_3_-Im)_2_

| **peak name** | **E_B_ / eV** | **area /cps eV** | **sens fact.** | **norm. area** | **quant. / at. %** |
| --- | --- | --- | --- | --- | --- |
| **C 1s** | **283.5** | **100508.6** | **18775** | **5.35307** | **53.02** |
| **N 1s** | **397.5** | **46408.44** | **34938** | **1.41416** | **14.01** |
| **O 1s** | **530.5** | **37038.17** | **57080** | **0.6488705** | **6.43** |
| **F 1s** | **685.5** | **181378.3** | **81508** | **2.225221** | **22.04** |
| **Co 2p** | **779.5** | **24205.16** | **326924** | **0.0740389** | **0.73** |
| **Zn 2p** | **1019.5** | **126121.2** | **330412** | **0.3817087** | **3.78** |

pyrolyzed qtz-Zn_0.9_Co_0.1_(CF_3_-Im)_2_

**Supplementary Figure 19:** XPS spectrum of pyrolyzed qtz-Zn_0.9_Co_0.1_(CF_3_-Im)_2_.

**Supplementary Table 10:** Analysis data of pyrolyzed qtz-Zn_0.9_Co_0.1_(CF_3_-Im)_2_

| **peak name** | **E_B_ / eV** | **area /cps eV** | **sens. fact.** | **norm. area** | **quant. / at. %** |
| --- | --- | --- | --- | --- | --- |
| **C 1s** | **282.5** | **262452.8** | **18766** | **13.98535** | **81.69** |
| **N 1s** | **396.5** | **52888** | **34926** | **1.514265** | **8.84** |
| **O 1s** | **529.5** | **67147.37** | **57111** | **1.175734** | **6.87** |
| **F 1s** | **685.5** | **15100.55** | **81508** | **0.1852626** | **1.08** |
| **Co 2p** | **778.5** | **74720.01** | **327144** | **0.2284006** | **1.33** |
| **Zn 2p** | **1019.5** | **10547.35** | **330412** | **0.0319217** | **0.19** |

pyrolyzed

qtz-Zn_0.9_Co_0.1_(CF_3_-Im)_2_

**Supplementary Figure 20:** XPS detail scan of the F-region showing organic (C-F) and inorganic (M-F) fluorine.

**Supplementary Table 11:** Analysis data of the F-region of pyrolyzed qtz-Zn_0.9_Co_0.1_(CF_3_-Im)_2_

| **peak name**  **F 1s** | **peak height / cps** | **lorentzian** | **position / eV** | **FWHM / eV** | **abs. area / cps eV** | **rel. area / %** |
| --- | --- | --- | --- | --- | --- | --- |
| **F inorganic** | **81.217** | **0.2** | **684.6968** | **1.97195** | **180.68** | **10.8** |
| **F organic** | **691.82** | **0.2** | **688.2581** | **1.90483** | **1493** | **89.2** |


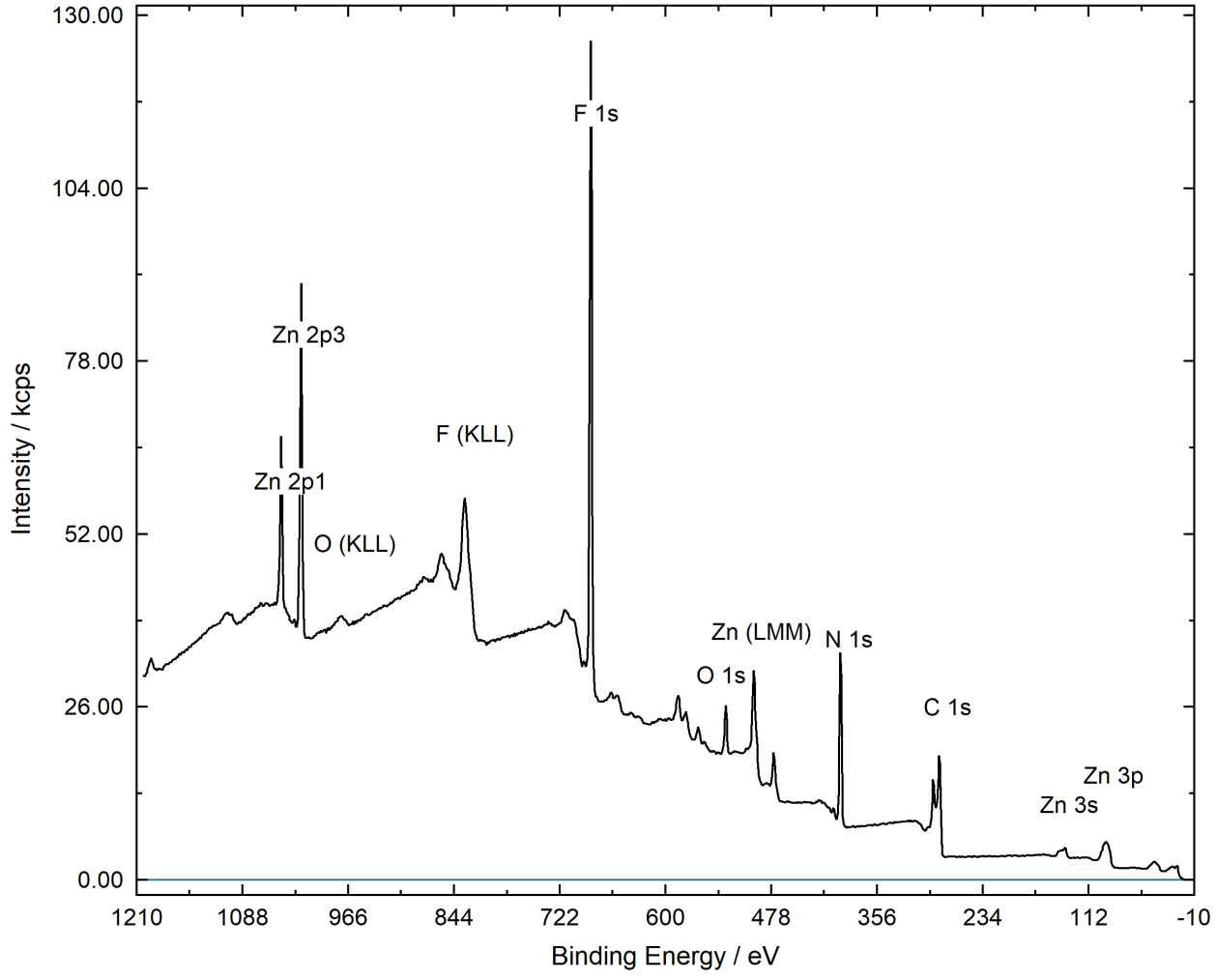


SOD-Zn(CF_3_-Im)_2_

**Supplementary Figure 21:** XPS spectrum of SOD-Zn(CF_3_-Im)_2_.

**Supplementary Table 12:** Analysis data of SOD-Zn(CF_3_-Im)_2_

| **peak name** | **E_B_ / eV** | **area /cps eV** | **sens. fact.** | **norm. area** | **quant. / at. %** |
| --- | --- | --- | --- | --- | --- |
| **C 1s** | **284.5** | **116957** | **18785** | **6.225928** | **46.38** |
| **N 1s** | **398.5** | **79057.56** | **34931** | **2.263213** | **16.86** |
| **O 1s** | **530.5** | **21294.26** | **57080** | **0.3730535** | **2.78** |
| **F 1s** | **686.5** | **309282** | **81479** | **3.795831** | **28.28** |
| **Zn 2p** | **1020.5** | **252392.9** | **329466** | **0.7660659** | **5.71** |

pyrolyzed SOD-Zn(CF_3_-Im)_2_

**Supplementary Figure 22:** XPS spectrum of pyrolyzed SOD-Zn(CF_3_-Im)_2_.

**Supplementary Table 13:** Analysis data of pyrolyzed SOD-Zn(CF_3_-Im)_2_

| **peak name** | **E_B_ / eV** | **area /cps eV** | **sens. fact.** | **norm. area** | **quant. / at. %** |
| --- | --- | --- | --- | --- | --- |
| **C 1s** | **282.5** | **223737.5** | **18766** | **11.92232** | **83.45** |
| **N 1s** | **396.5** | **37434.16** | **34926** | **1.071797** | **7.5** |
| **O 1s** | **529.5** | **52600.72** | **57111** | **0.9210258** | **6.45** |
| **F 1s** | **686.5** | **15078.01** | **81508** | **0.1849861** | **1.29** |
| **Zn 2p** | **1018.5** | **61867.45** | **331028** | **0.1868948** | **1.31** |

pyrolyzed

SOD-Zn(CF_3_-Im)_2_

**Supplementary Figure 23:** XPS spectrum of the F-region of pyrolyzed SOD-Zn(CF_3_-Im)_2_.

**Supplementary Table 14:** Analysis data of the F-region of pyrolyzed SOD-Zn(CF_3_-Im)_2_

| **peak name**  **F 1s** | **peak height / cps** | **lorentzian** | **position / eV** | **FWHM / eV** | **abs. area / cps eV** | **rel. area / %** |
| --- | --- | --- | --- | --- | --- | --- |
| **F inorganic** | **15.742** | **0.2** | **684.6031** | **1.8** | **31.86** | **2.09** |
| **F organic** | **717.276** | **0.2** | **688.158** | **1.83139** | **1492** | **97.91** |

SOD-Zn_0.9_Co_0.1_(CF_3_-Im)_2_

**Supplementary Figure 24:** XPS spectrum of SOD-Zn_0.9_Co_0.1_(CF_3_-Im)_2_.

**Supplementary Table 15:** Analysis data of SOD-Zn_0.9_Co_0.1_(CF_3_-Im)_2_

| **peak name** | **E_B_ / eV** | **area /cps eV** | **sens. fact.** | **norm. area** | **quant. / at. %** |
| --- | --- | --- | --- | --- | --- |
| **C 1s** | **290.5** | **66893.62** | **18824** | **3.553625** | **45.41** |
| **N 1s** | **397.5** | **42040.8** | **34938** | **1.203285** | **15.38** |
| **O 1s** | **530.5** | **22325.57** | **57080** | **0.391121** | **5** |
| **F 1s** | **686.5** | **182927.2** | **81479** | **2.245074** | **28.69** |
| **Co 2p** | **780.5** | **43387.76** | **326465** | **0.1329016** | **1.7** |
| **Zn 2p** | **1020.5** | **98501.4** | **329466** | **0.2989726** | **3.82** |

pyrolyzed SOD-Zn_0.9_Co_0.1_(CF_3_-Im)_2_

**Supplementary Figure 25:** XPS spectrum of pyrolyzed SOD-Zn_0.9_Co_0.1_(CF_3_-Im)_2_.

**Supplementary Table 16:** Analysis data of pyrolyzed SOD-Zn_0.9_Co_0.1_(CF_3_-Im)_2_

| **peak name** | **E_B_ / eV** | **area /cps eV** | **sens. fact.** | **norm. area** | **quant. / at. %** |
| --- | --- | --- | --- | --- | --- |
| **C 1s** | **284.5** | **186161.9** | **18785** | **9.909886** | **81.64** |
| **N 1s** | **398.5** | **31168.33** | **34931** | **0.8922683** | **7.35** |
| **O 1s** | **531.5** | **57873.34** | **57084** | **1.013824** | **8.35** |
| **F 1s** | **687.5** | **10269.59** | **81395** | **0.1261693** | **1.04** |
| **Co 2p** | **779.5** | **58982.34** | **326924** | **0.1804156** | **1.49** |
| **Zn 2p** | **1020.5** | **5201.787** | **329466** | **0.0157885** | **0.13** |

pyrolyzed

SOD-Zn_0.9_Co_0.1_(CF_3_-Im)_2_

**Supplementary Figure 26:** XPS spectrum of the F-region of pyrolyzed SOD-Zn_0.9_Co_0.1_(CF_3_-Im)_2_.

**Supplementary Table 17:** Analysis data of the F-region of pyrolyzed SOD-Zn_0.9_Co_0.1_(CF_3_-Im)_2_

| **peak name**  **F 1s** | **peak height / cps** | **lorentzian** | **position / eV** | **FWHM / eV** | **abs. area / cps eV** | **rel. area / %** |
| --- | --- | --- | --- | --- | --- | --- |
| **F inorganic** | **67.909** | **0.2** | **684.9224** | **1.96619** | **153.02** | **12.19** |
| **F organic** | **485.826** | **0.2** | **688.2847** | **1.98225** | **1103** | **87.81** |
